# Temperature-controlled conformational switching of squaramides enabled by side-chain hydrogen bonding

**DOI:** 10.1039/d6ra06416g

**Published:** 2026-08-04

**Authors:** Kazusa Kuyama, Kimiko Tanaka, Fumi Takeda, Sakura Nakagawa, Shiho Ohno, Yoshiki Yamaguchi, Kosuke Katagiri, Masatoshi Kawahata, Isao Azumaya, Iwao Okamoto, Hiroyuki Kagechika, Aya Tanatani

**Affiliations:** a Department of Chemistry, Faculty of Science, Ochanomizu University Tokyo Japan tanatani.aya@ocha.ac.jp; b Institute of Molecular Biomembrane and Glycobiology, Tohoku Medical and Pharmaceutical University Miyagi Japan; c Department of Chemistry of Functional Molecules, Faculty of Science and Engineering, Konan University Hyogo Japan; d Faculty of Pharmaceutical Science, Showa Pharmaceutical University Tokyo Japan; e Faculty of Pharmaceutical Sciences, Toho University Chiba Japan; f Laboratory for Biomaterials and Bioengineering, Institute of Integrated Research, Institute of Science Tokyo Tokyo Japan; g International Institute for Sustainability with Knotted Chiral Meta Matter (WPI-SKCM2), Hiroshima University Hiroshima Japan

## Abstract

Precise control of local conformational bias is essential for constructing stimulus-responsive aromatic architectures. Amide-derived scaffolds such as squaramides are particularly attractive targets, as their moderate rotational barriers allow thermodynamic tuning without irreversible locking. Here we demonstrate that introduction of a hydrogen-bond-accepting side chain enables thermodynamic programming of squaramide conformations. The triethylene glycol (TEG) substituent of squaramide 1c bearing a pyrene moiety induces reversible temperature-dependent switching between (*trans*, *trans*) and (*cis*, *trans*) forms, which can be quantified by variable-temperature NMR and van't Hoff analysis (Δ*H*° = −1.87 kcal mol^−1^, Δ*S*° = −8.2 cal mol^−1^ K^−1^). In contrast, *N-n*-propyl analogue 1b lacking a hydrogen-bond-accepting functionality exists exclusively in (*cis*, *trans*) form under identical conditions, establishing the minimal structural requirement for switching. A diphenyl analogue 1d without π-extension retains thermally reversible behavior, demonstrating that π-surface enlargement is not essential, but modulates conformational bias. These results establish a side-chain-enabled design principle for thermodynamically programmable aromatic conformations.

## Abstract 2

Precise control of local conformational bias is essential for constructing stimulus-responsive aromatic architectures.

## Graphical abstract

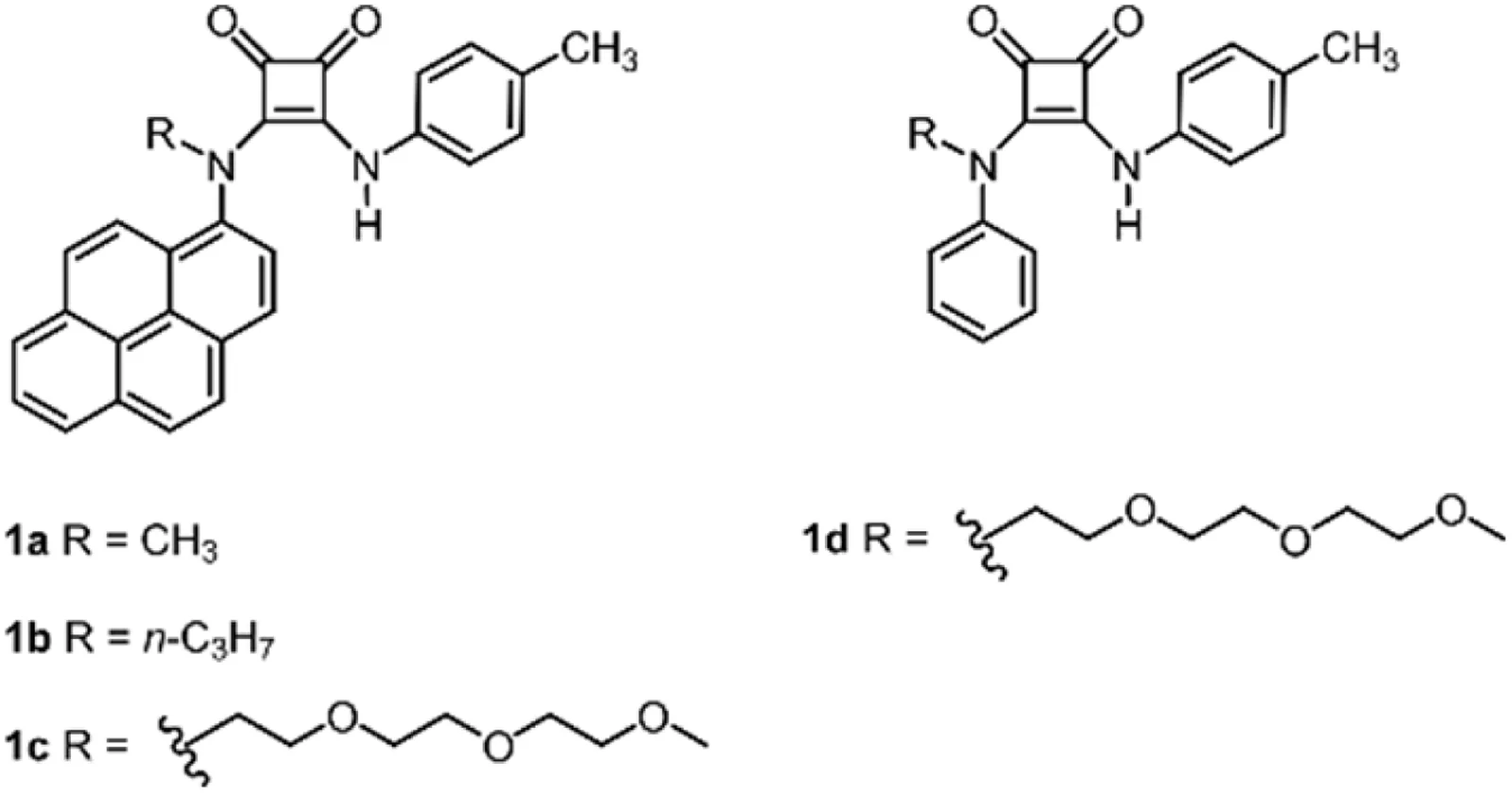

## Introduction

Organic molecules often exist as multiple geometric or conformational isomers that exhibit distinct chemical and physical properties. The ability to reversibly control such isomeric equilibria in response to external stimuli is a fundamental requirement for constructing dynamic molecular systems. While olefins and azo derivatives have long served as prototypical platforms for photoinduced switching, their large rotational barriers limit thermally accessible interconversion.^1–10^

In contrast, amide-derived scaffolds possess moderate rotational barriers (typically 15–20 kcal mol^−1^), enabling thermodynamically tunable conformational equilibria that can be probed by dynamic NMR spectroscopy.^11^ This balance between structural rigidity and accessibility makes amide-based systems attractive candidates for stimulus-responsive conformational control. For example, we observed solvent-dependent conformational switching of some aromatic amide systems, which suggests that local interactions at the nitrogen substituent can significantly influence equilibrium populations.^12–14^

Among amide-related compounds, squaramides are of particular interest owing to their conjugated four-membered core and strong hydrogen-bonding capability.^15–21^ Like aromatic amides and ureas,^22–24^ aromatic squaramides can adopt distinct *cis*–*trans* conformations, and *N*-substitution has been shown to influence their conformational preference. ^25^ Thus, *N*,*N*′-diphenylsquaramides existed in (*trans*, *trans*) conformation, while introduction of *N*-monomethyl or *N*,*N*′-dimethyl groups changed the preferred conformation to (*trans*, *cis*) or (*cis*, *cis*), respectively.^26^ However, rational strategies to thermodynamically regulate squaramide conformational equilibria remain underexplored.

In our previous study, among *N*,*N*′-diarylsquaramides, only *N*-monomethylated compounds exhibited unique solvent-sensitive conformational bias.^27^ During our systematic investigation on the solvent-dependent properties of *N*-monoalkylated squaramide derivatives, we found that the squaramide derivatives with a hydrogen-bond-accepting side chain not only changed in a solvent-dependent manner, but also exhibits a unique property of changing in a temperature-dependent manner.

## Results and discussion

**Fig. 1:**
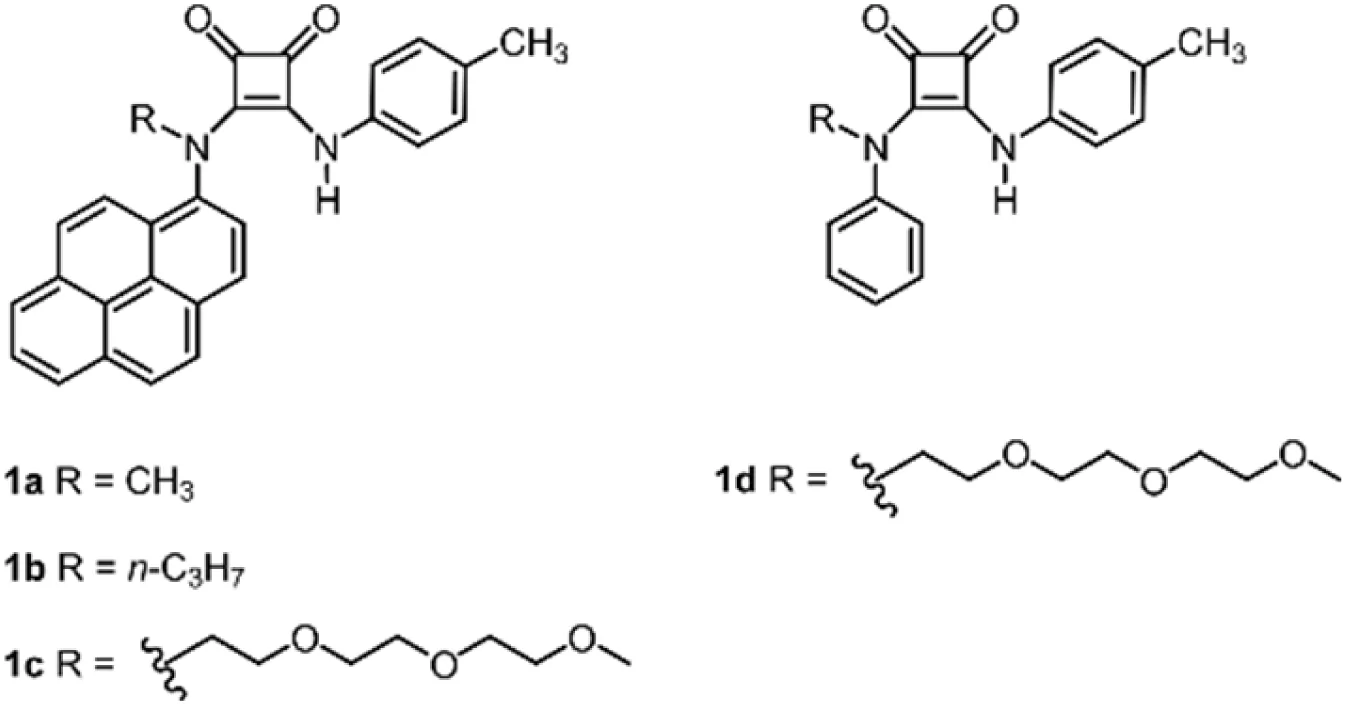
Structures of squaramides 1a–1d.

To examine the conformational behavior of aromatic squaramides, four derivatives 1a–1d were prepared (Fig. 1). The *N*-substituent of *N-*(1-pyrenyl)-*N-p*-tolylsquaramide was systematically varied from methyl (1a), to *n*-propyl (1b), to triethylene glycol (1c), while the *N*-aromatic unit was changed to a phenyl group (1d). Compounds 1a–1c were synthesized *via* stepwise substitution of diethyl squarate with the corresponding secondary aminopyrene 3a–3c, respectively, in the presence of zinc triflate as a Lewis acid catalyst, followed by reaction with *p*-toluidine (Scheme S1). The phenyl analogue 1d was prepared under analogous conditions using aniline instead of the aminopyrene (Scheme S2). All compounds were obtained in moderate to good yields after purification by column chromatography.

**Fig. 2:**
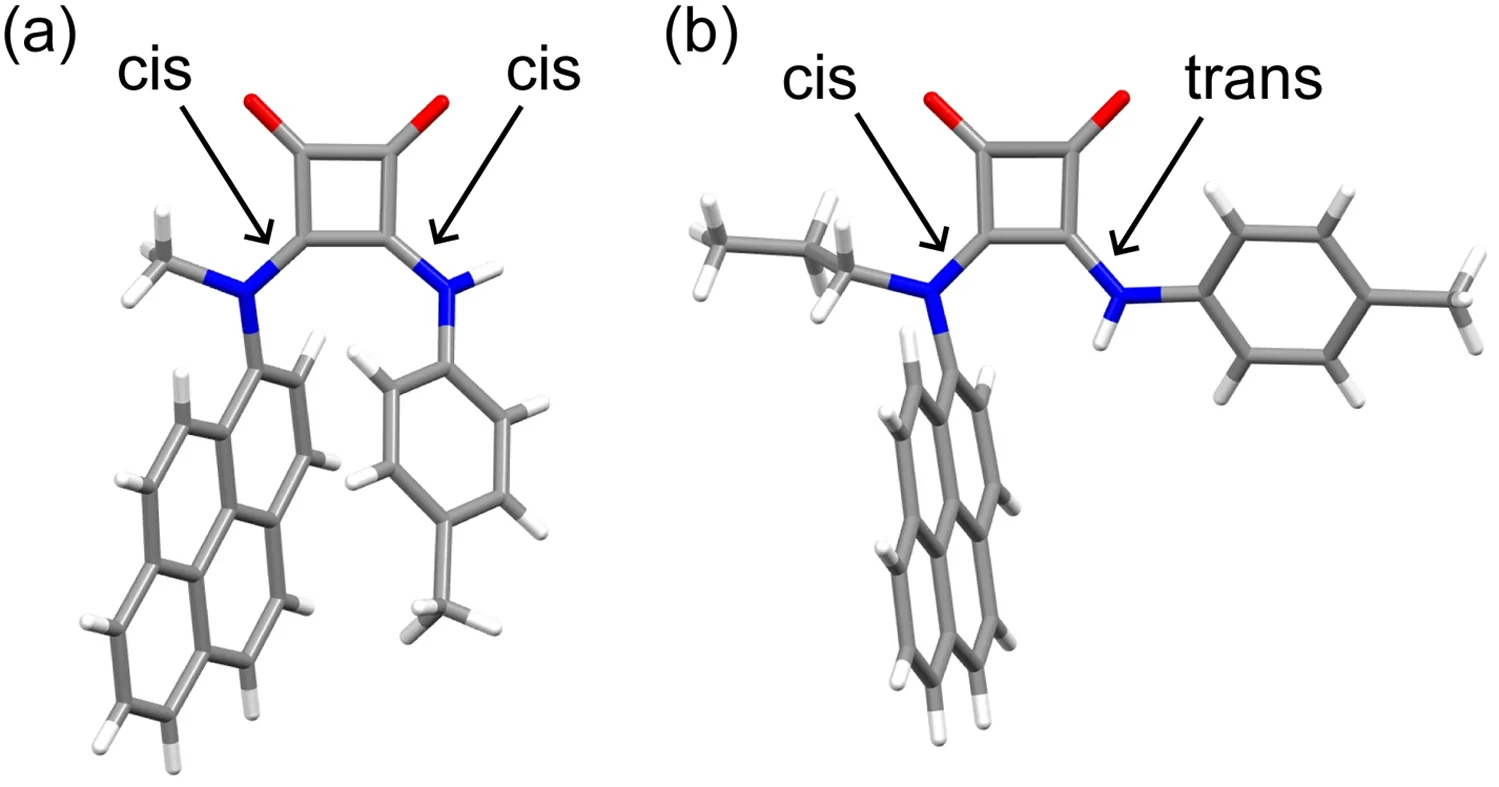
Crystal structures of squaramides (a) 1a (CCDC: 2541946) and (b) 1b (CCDC: 2541947).

X-Ray crystallographic analysis revealed that compound 1b bearing an *N-n*-propyl group adopts a (*cis*, *trans*) conformation in the solid state, with the *N*-propylated side in the *cis* arrangement, as anticipated (Fig. 2 and Table S1). In the crystal packing, two molecules of 1b form a discrete dimer through π–π interactions between the pyrenyl and tolyl rings, accompanied by additional C–H⋯π contacts involving the pyrene moieties (Fig. S1b). In contrast, the crystal structure of 1a showed a (*cis*, *cis*) conformation (Fig. 2). In this structure, intermolecular hydrogen bonds are formed between the NH group of one molecule and the carbonyl oxygen of the squaramide unit of another, resulting in a hydrogen-bonded assembly (Fig. S1a). Comparison of these crystal structures clearly indicates that the size of the *N*-alkyl substituent significantly influences the conformational preference in the solid state. The smaller methyl group in 1a allows a close molecular approach, facilitating intermolecular hydrogen bonding. By contrast, the bulkier propyl group in 1b prevents such proximity due to steric hindrance, favoring π–π interactions between aromatic rings instead.

**Fig. 3:**
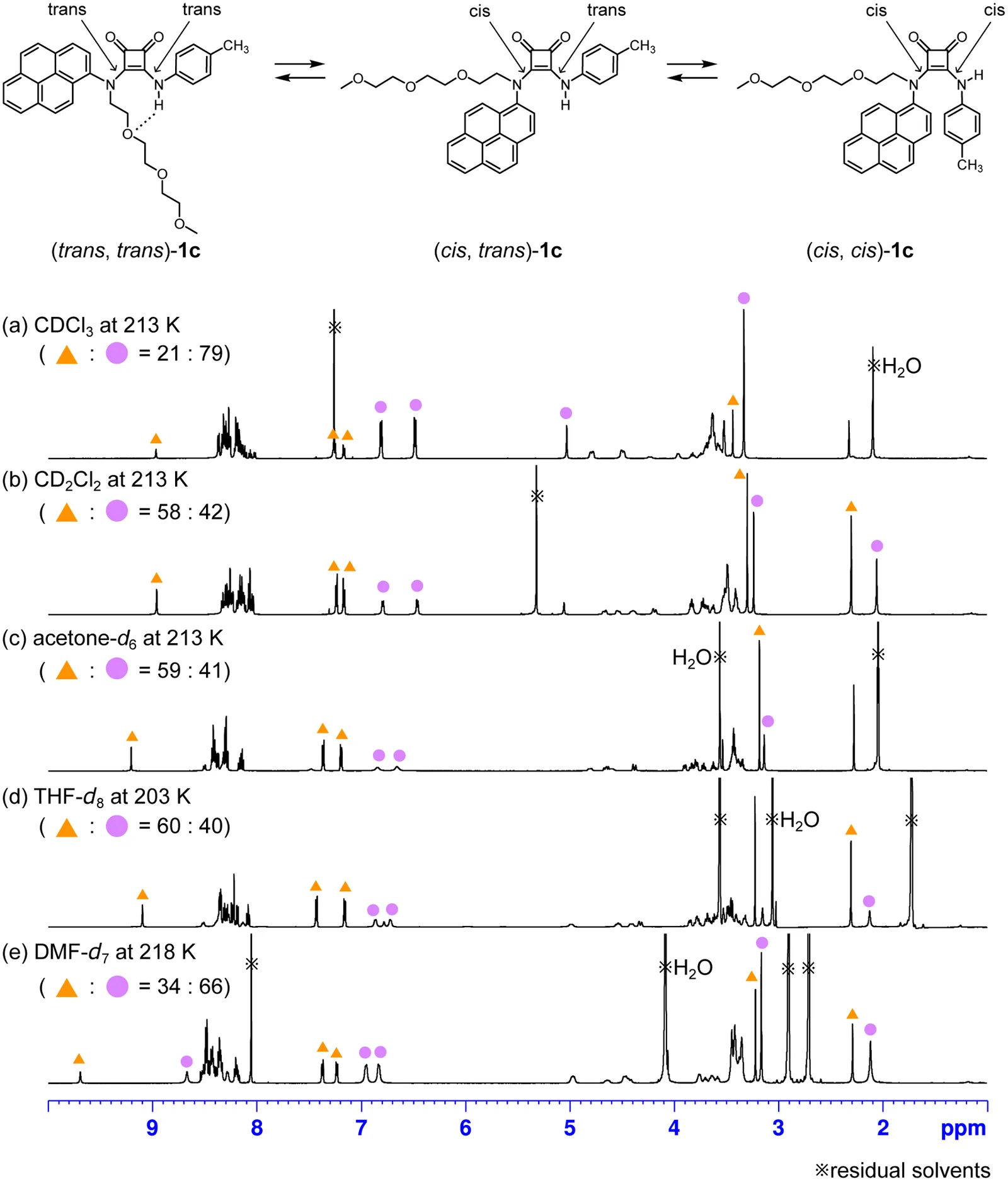
Solvent-dependent ^1^H NMR spectra of 1c in (a) CDCl_3_, (b) CD_2_Cl_2_, (c) acetone-*d*_6_, (d) THF-*d*_8_, and (e) DMF-*d*_7_ (600 MHz, 10 mM).

In solution, 1a and 1b both exist in the (*cis*, *trans*) form (>99.9%) in CD_2_Cl_2_, and no other conformer was observed even at 183 K (Fig. S2). To improve the limited solubility of 1a and 1b, compound 1c was designed and synthesized by introducing a triethylene glycol (TEG) chain on the nitrogen atom, as shown in Scheme S1. The conformational behavior of 1c was investigated by variable-temperature ^1^H NMR spectroscopy. Interestingly, two distinct conformers were observed in solution, and their relative populations varied depending on the solvent, as shown in Fig. 3.

The conformational equilibrium of 1c was found to be strongly solvent-dependent. In CD_2_Cl_2_ at 213 K, two conformers were observed in a ratio of 58 : 42 (Table 1). Similar distributions were obtained in acetone-*d*_6_ (59 : 41) and THF-*d*_8_ (60 : 40). In contrast, the population was reversed in CDCl_3_, with the alternative conformer becoming predominant (21 : 79), and a similar tendency was observed in DMF-*d*_7_ (34 : 66). These results indicate that the two conformers are nearly isoenergetic and thus subtle differences in solvent–solute interactions can significantly influence the conformational equilibrium.

**Table 1:** Gibbs' free energy difference and the rotation barrier of 1c in various solvents^a^

Solvent	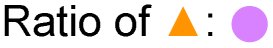	*T* [K]	Δ*G*° [kcal mol^−1^]	*T*_c_	Δ*v* [Hz]	Δ*G*^‡^ [kcal mol^−1^]
CDCl_3_	21 : 79	213	0.56	283	168.0^a^	13.19
CD_2_Cl_2_	58 : 42	213	−0.14	296	147.0^a^	13.90
Acetone-*d*_6_	59 : 41	213	−0.15	283	201.8^a^	13.08
THF-*d*_8_	60 : 40	203	−0.16	253	13.4^b^	13.00
DMF-*d*_7_	34 : 66	218	0.29	283	191.6^a^	13.11

a Δ*ν* was determined from the signal of (a) methyl protons on tolyl group or (b) *N*-methyl protons at *T*_c_.

The conformer assignment was first inferred from a comparison with the chemical shifts of 1a and 1b, which exist exclusively in the (*cis*, *trans*) form in various solvents. The minor conformer of 1c in CD_2_Cl_2_ exhibited similar aromatic chemical shifts and was therefore tentatively assigned as the (*cis*, *trans*) form. The alternative conformer showed a markedly downfield-shifted NH signal, suggesting the presence of intramolecular hydrogen bonding between the squaramide NH and the ether oxygen atoms of the TEG chain and thus indicating a (*trans*, *trans*) arrangement. The assignment of the (*cis*, *trans*) form was further supported by NOESY measurements of 1c in CD_2_Cl_2_ at 183 K. As shown in Fig. S3, cross peaks were observed between the NH proton and the pyrenyl protons, as well as between the ortho protons of the tolyl group and the pyrenyl moiety. These spatial correlations indicate close proximity between the squaramide NH, the tolyl ring, and the pyrene unit, consistent with (*cis*, *trans*) conformation.

To further substantiate the conformational assignment, density functional theory (DFT) calculations were performed for the four possible conformers of 1c: (*cis*, *cis*), (*cis*, *trans*), (*trans*, *cis*) and (*trans*, *trans*). In addition, an extensive conformational search was conducted, generating 20 736 random conformers, from which the energies corresponding to these four representative conformers were extracted. The relative energies of the (*cis*, *cis*), (*cis*, *trans*), (*trans*, *cis*) and (*trans*, *trans*) conformers were 0.0 (reference), −4.7, 5.7 and −4.0 kcal mol^−1^, respectively. Comparison of the calculated chemical shifts and relative energies with the experimental data confirmed that the two observed species correspond to the (*cis*, *trans*) and (*trans*, *trans*) conformers (Fig. S4). Accordingly, the (*cis*, *trans*) conformer was predominant in CDCl_3_ and DMF-*d*_7_, while (*trans*, *trans*) conformer was predominant in CD_2_Cl_2_, acetone-*d*_6_, and THF-*d*_8_. At present, the cause of this solvent-dependent conformational change is unclear, while it is thought to be due to difference in the hydrogen-bonding ability of the TEG substituent in each solvent, since this characteristic was observed only in 1c and not in 1a and 1b.

**Fig. 4:**
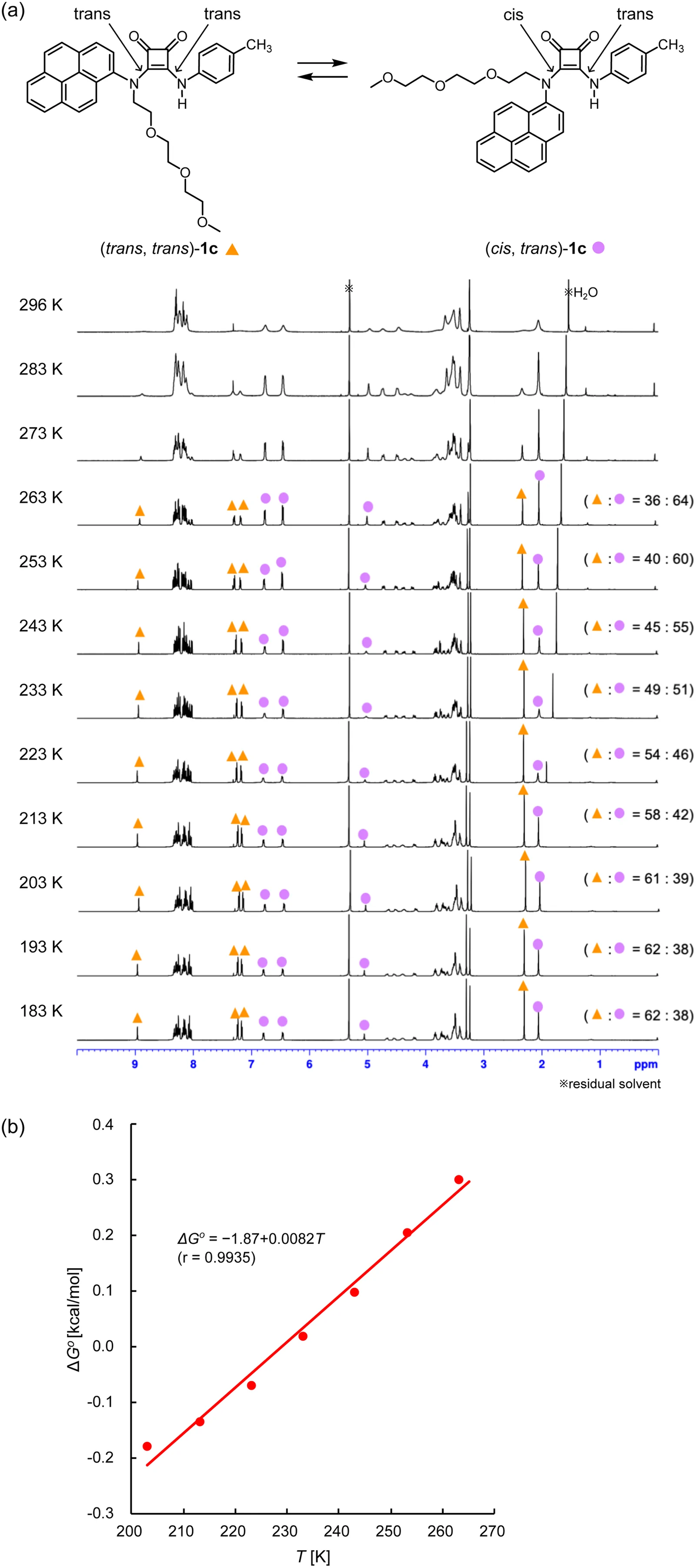
(a) Temperature-dependent ^1^H NMR spectra of 1c in CD_2_Cl_2_ and (b) van't Hoff plot (600 MHz, 10 mM).

Variable-temperature ^1^H NMR experiments of 1c in CD_2_Cl_2_ revealed a clear temperature dependence of the conformational equilibrium (Fig. 4a). At low temperature (183 K), the (*trans*, *trans*) conformer was predominant (62 : 38). Upon increasing the temperature, the population of the (*cis*, *trans*) conformer gradually increased, and a reversal of the conformer ratio was observed around 233 K. At 263 K, the ratio reached 36 : 64.

This behavior suggests that the (*trans*, *trans*) conformer is enthalpically stabilized at lower temperature, most likely through intramolecular hydrogen bonding between the squaramide NH and the ether oxygen atoms of the TEG chain, whereas the (*cis*, *trans*) conformer becomes favored at higher temperature due to entropic contributions. To quantify the thermodynamic parameters governing the conformational equilibrium, Δ*G*° values were calculated from the temperature-dependent population ratios and plotted against temperature (Fig. 4b). Linear fitting afforded the following relationship: Δ*G*° = −1.87 + 0.0082*T* (kcal mol^−1^).

From this analysis, Δ*H*° and Δ*S*° were determined to be −1.87 kcal mol^−1^ and −8.2 cal mol^−1^ K^−1^, respectively. The negative Δ*H*° value indicates that the (*trans*, *trans*) conformer is enthalpically favored, consistent with stabilization by intramolecular hydrogen bonding between the squaramide NH and the ether oxygen atoms of the TEG chain. In contrast, the negative Δ*S*° value suggests that this conformer is entropically disfavored due to the conformational restriction imposed by hydrogen-bond formation. Consequently, increasing the temperature favors the (*cis*, *trans*) conformer, leading to the observed reversal of the conformational ratio.

Similar temperature-dependent changes were observed in acetone-*d*_6_ (Fig. S5). At low temperature, the (*trans*, *trans*) conformer was predominant, whereas increasing the temperature shifted the equilibrium toward the (*cis*, *trans*) form. This consistent behavior in two solvents supports the proposed thermally controlled conformational switching of 1c.

To clarify the role of π-extension in this thermodynamic switching, the phenyl analogue 1d was examined under identical conditions (Fig. S6). Two conformers were clearly resolved below the coalescence temperature (223 K) in CD_2_Cl_2_, allowing determination of Δ*G*° values in this temperature range. Linear fitting of Δ*G*° as a function of temperature afforded Δ*H*° = −1.79 kcal mol^−1^ and Δ*S*° = −8.0 cal mol^−1^ K^−1^, similar to the corresponding values of 1c. Thus, π-extension is not required for temperature-controlled conformational switching. The extended aromatic surface therefore appears to act primarily as a modulating factor rather than an essential structural element for the thermodynamic regulation.

The contrasting NMR behavior of 1b and 1c provides strong support for the role of the TEG chain in the conformational switching observed for 1c. Under identical experimental conditions (CD_2_Cl_2_, 183 K, 10 mM), 1c exhibited two distinct sets of signals corresponding to two conformers in equilibrium, whereas 1b showed only a single set of signals (Fig. S2b). No additional signals attributable to a (*trans*, *trans*) conformer were detected for 1b within the NMR detection limit.

Based on a comparison of the chemical shifts with those of 1a, 1b was assigned to adopt exclusively the (*cis*, *trans*) form in solution over the examined temperature range. In contrast, 1c displays a temperature-dependent equilibrium between the (*cis*, *trans*) and (*trans*, *trans*) forms. Because the *N-n*-propyl substituent in 1b lacks a hydrogen-bond acceptor, whereas the TEG chain in 1c contains multiple ether oxygen atoms capable of interacting with the squaramide NH, these observations are consistent with intramolecular hydrogen bonding in 1c stabilizing the (*trans*, *trans*) conformer at low temperature. Thus, 1b serves as an effective negative-control analogue, supporting the conclusion that the conformational switching observed in 1c originates from the hydrogen-bonding capability introduced with the TEG side chain.

The present results show that introduction of a hydrogen-bond-accepting side chain enables temperature-dependent control of squaramide conformational equilibria. The balance between enthalpic stabilization by intramolecular hydrogen bonding and entropic contributions governs the reversible interconversion between distinct conformational states. This thermodynamically regulated local conformational bias may serve as a useful element in the design of stimulus-responsive aromatic systems.

## Conclusions

We have demonstrated that introduction of a hydrogen-bond-accepting side chain enables temperature-controlled conformational switching in squaramides. The triethylene glycol substituent stabilizes the (*trans*, *trans*) form of 1c enthalpically through intramolecular hydrogen bonding, whereas entropic contributions favor the (*cis*, *trans*) form at elevated temperature, leading to a reversible thermodynamic interconversion. The absence of switching in the *N-n*-propyl analogue 1b establishes the essential role of the side-chain hydrogen-bond acceptor, while the phenyl analogue confirms that π-extension is not required, but serves to modulate the conformational bias. The moderate stabilization of intramolecular hydrogen bond formation with an eight-membered ring gives rise to temperature-dependent conformational change. Although the design of compounds exhibiting a greater temperature-dependent conformational bias would be desirable, these findings provide a framework for the rational control of local aromatic conformation in response to thermal stimuli.

## Author contributions

H. K. and A. T. refined and adapted overarching research goals and aims. K. Kuyama and K. T. synthesized, purified and characterized the compounds. K. Kuyama, K. T. and I. A. examined the spectral data. S. O. and Y. Y. examined the theoretical studies. K. Katagiri, M. K. and I. O. examined the crystallographic analysis. F. T. and S. N. provided comments and prepared the figures and tables for the draft. All authors approved the final manuscript.

## Conflicts of interest

There are no conflicts to declare.

## Supplementary Material

RA-016-D6RA06416G-s001

RA-016-D6RA06416G-s002

## Data Availability

CCDC 2541946 and 2541947 contain the supplementary crystallographic data for this paper.^30*a*,*b*^ Ref. 28 and 29 are cited in the supplementary information (SI). The data supporting this article have been included as part of the SI. Supplementary information: synthetic protocol, NMR spectra, crystallographic information and theoretical computational data. See DOI: https://doi.org/10.1039/d6ra06416g.
