# Supplementary material for: Temperature-controlled conformational switching of squaramides enabled by side-chain hydrogen bonding
Source: RSC Adv. 2026 Aug 4;16(41):48015–9. doi: 10.1039/d6ra06416g (PMC13436565; doi:10.1039/d6ra06416g)
Supplement: Supplementary file 1 [file RA-016-D6RA06416G-s001.pdf]

Supplementary Information (SI) for RSC Advances.
This journal is © The Royal Society of Chemistry 2026

Supporting Information

Temperature-Controlled Conformational Switching of Squaramides Enabled by Side-Chain Hydrogen Bonding

Kazusa Kuyama, Kimiko Tanaka, Fumi Takeda, Shiho Ohno, Yoshiki Yamaguchi, Kosuke Katagiri, Masatoshi Kawahata, Isao Azumaya, Iwao Okamoto, Hiroyuki Kagechika, Aya Tanatani

Contents **page**

1. Experimental
  1.1. General S2
  1.2. Synthesis of compounds **1a** – **1d** (Scheme S1 and S2) S2
  1.3. ^{1}H and ^{13}C{^{1}H} NMR charts of compounds **1a** – **1d** S8

2. X-Ray Crystallographic analysis of compounds **1a** and **1b** (Table S1) S12

3. Crystal Packing structures of compounds **1a** and **1b** (Figure S1) S13

4. Temperature-dependent ^{1}H NMR spectra of compounds **1a** and **1b** (Figure S2) S14

5. NMR spectra and computational details of compound **1c**
  4.1. NOESY spectra (Figure S3) S15
  4.2. Computational methods and cartesian coordinates (Figure S4) S16
  4.3. Temperature-dependent ^{1}H NMR spectra (Figure S5) S21

6. Temperature-dependent ^{1}H NMR spectra of compound **1d** (Figure S6) S22

1. Experimental

1.1. General

All reagents were purchased from Sigma-Aldrich Chemical Co., Wako Pure Chemical Industries, Tokyo Kasei Kogyo Co., and Kanto Kagaku Co., Inc. Silica Gel 60 N (spherical, neutral) for column chromatography was purchased from Kanto Kagaku Co., Inc. ^{1}H and ^{13}C NMR spectra were recorded on Bruker Avance II 600, Bruker Avance III 600, and JEOL ECZL 500R spectrometers with chloroform (7.26 ppm), methylene chloride (5.32 ppm), methanol (3.31 ppm), DMSO (2.50 ppm), acetone (2.05 ppm), DMF (2.74 ppm) or THF (1.73 ppm) as the internal standard. Chemical shifts are reported in parts per million (ppm, δ) relative to the signal of the NMR solvent used. ^{1}H NMR splitting patterns with observed first-order coupling are designated as singlet (s), doublet (d), triplet (t), or quartet (q). Coupling constants (J) are reported in hertz. Splitting patterns that could not be interpreted or easily visualized are designated as multiplet (m) or broad (br). Mass spectral data were obtained on a Bruker compact ESI-QTOF in the positive/negative ion detection mode.

1.2. Synthesis of compounds 1a–1d

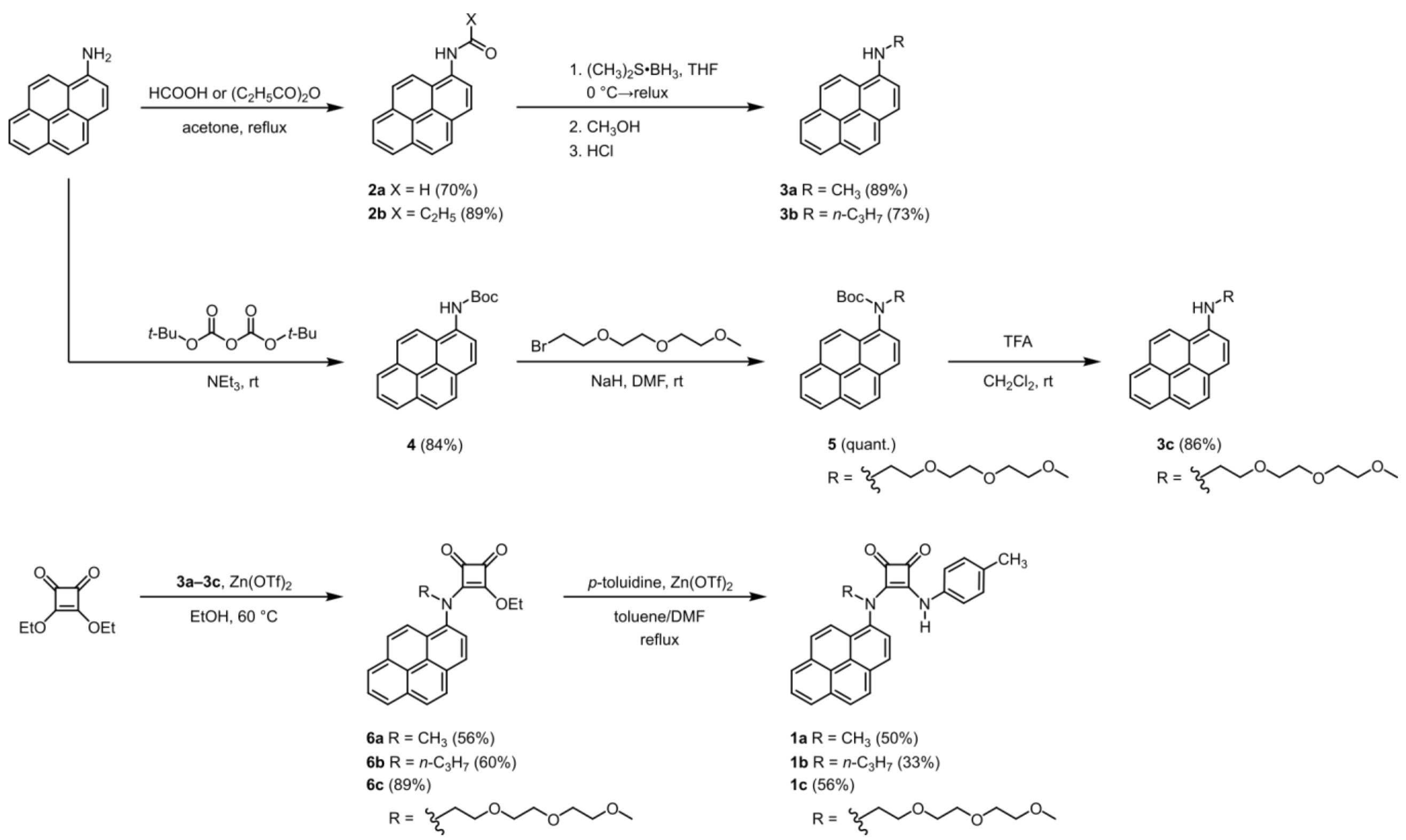

Scheme S1. Synthetic scheme for compounds **1a** – **1c**

Synthesis of **2a**. 1-Aminopyrene (254.7 mg, 1.20 mmol) was added to formic acid 7.5 mL, and the mixture was heated at 75 ℃ for 4 h. After removal of the solvent under vacuum, the residue was extracted with ethyl acetate and water. The organic layer was dried over magnesium sulfate, filtered and evaporated. The residue was washed by chloroform to give **2a** (206.1 mg, 70%) as white powder. Compound **2a**: m.p. 221–224 ℃; ^{1}H NMR (600 MHz, DMSO-d_6) δ 10.70 (br s, 1H), 8.60 (d, 1H, J = 1.8 Hz), 8.56 (d, 1H, J = 8.4 Hz), 8.38 (d, 1H, J = 9.6 Hz), 8.31–8.28 (m, 3H), 8.25 (d, 1H, J = 9.0 Hz), 8.18–8.13 (m, 2H) , 8.09 (t, 1H, J = 7.2 Hz); ^{13}C NMR (150 MHz, DMSO-d_6) δ 160.4, 130.9, 130.8, 130.4, 127.8, 127.23,

127.16, 126.37, 126.35, 125.2, 125.0, 124.8, 124.3, 123.8, 122.0, 121.4, 121.2; HRMS (ESI−) m/z: calcd for $C_{17}H_{19}NO$ $[M-H]^-$ 244.0768; found 244.0769.

Synthesis of **2b**. A solution of 1-aminopyrene (152.7 mg, 0.702 mmol) in acetone 1 mL was added to propionic anhydride (188.8 mg, 1.45 mmol) in acetone 3 mL, and the mixture was heated at 80 ℃ for 4 h. Cooling to room temperature afforded a white precipitate, which was collected and washed with acetone to give **2b** (170.7 mg, 0.625 mmol, 89%) as white powder. Compound **2b**: m.p. 218 ℃; ^{1}H NMR (600 MHz, DMSO-d_6) δ 10.22 (br, s, 1H), 8.29–8.23 (m, 5H), 8.20 (d, 1H, *J* = 9.0 Hz), 8.14 (dd, 2H, *J* = 9.0, 8.4 Hz), 8. 06 (t, 1H, *J* = 7.8 Hz), 2.56 (q, 2H, *J* = 7.2 Hz), 1.22 (t, 3H, *J* = 7.2 Hz); ^{13}C NMR (150 MHz, DMSO-d_6) δ 173.3, 132.5, 131.3, 131.0, 128.5, 127.7, 127.4, 126.9, 126.8, 125.6, 125.4, 125.3, 124.8, 124.4, 124.2, 123.8, 122.9, 29.6, 10.4; HRMS (ESI+) m/z: calcd for $C_{19}H_{16}NO$ $[M+H]^+$ 274.1226; found 274.1226.

Synthesis of **3a**. Boran-methyl sulfide complex (0.8 mL, 8.43 mmol) was added slowly to a solution of **2a** (101.2 mg, 0.41 mmol) in THF (30 mL) at 0 ℃ under Ar atmosphere, and the mixture was heated at reflux for 3 h. After cooling to room temperature, methanol 3 mL was added at 0 ℃ and the mixture was stirred for 1 h. Then concentrated hydrochloric acid 3 mL was added and the mixture was stirred for 1 h. After the removal of the solvent under vacuum, a small portion of water was added to the residue. The solution was neutralized with saturated aqueous sodium bicarbonate (pH>7), and extracted with ethyl acetate. The organic layer was dried over magnesium sulfate, filtered and evaporated. The residue was purified by silica gel column chromatography (*n*-hexane/ethyl acetate 20:1) to give **3a** (84.5 mg, 89%) as green powder. Compound **3a**: m.p. 63–64 ℃; ^{1}H NMR (600 MHz, DMSO-d_6) δ 8.29 (d, 1H, *J* = 9.0 Hz), 8.07 (d, 1H, *J* = 8.4 Hz), 8.00–7.94 (m, 3H), 7.89–7.86 (m, 2H), 7.69 (d, 1H, *J* = 8.4 Hz), 7.23 (d, 1H, *J* = 8.4 Hz), 6.85 (q, 1H, *J* = 4.8 Hz) , 3.00 (d, 3H, *J* = 4.8 Hz); ^{13}C NMR (150 MHz, DMSO-d_6) δ 144.6, 132.1, 131.5, 127.8, 126.8, 125.9, 125.4, 125.1, 124.5, 122.9, 122.5, 121.8, 121.2, 121.0, 115.3, 107.7, 30.2; HRMS (ESI+) m/z: calcd for $C_{17}H_{14}N$ $[M+H]^+$ 232.1121; found 232.1123.

Synthesis of **3b**. Boran-methyl sulfide complex (200 µL, 2.11 mmol) was added slowly to a solution of **2b** (148.3 mg, 0.542 mmol) in THF (14 mL) at 0 ℃ under Ar atmosphere, and the mixture was heated at reflux for 4 h. After cooling to room temperature, methanol 8 mL was added at 0 ℃ and the mixture was stirred for 1 h. Then concentrated hydrochloric acid 8 mL was added and the mixture was stirred for 3 h. After the removal of the solvent under vacuum, a small portion of water was added to the residue. The solution was neutralized with saturated aqueous sodium bicarbonate (pH>7), and extracted with ethyl acetate. The organic layer was dried over magnesium sulfate, filtered and evaporated. The residue was purified by silica gel column chromatography (*n*-hexane/ethyl acetate 10:1) to give **3b** (102.7 mg, 0.396 mmol, 73%) as green powder. Compound **3b**: m.p. 158–160 ℃; ^{1}H NMR (600 MHz, DMSO-d_6) δ 8.37 (d, 1H, *J* = 9.0 Hz), 8.05 (d, 1H, *J* = 8.4 Hz), 8.01–7.94 (m, 3H), 7.87 (dd, 2H, *J* = 9.0, 7.8 Hz), 7.69 (d, 1H, *J* = 9.0 Hz), 7.30 (d, 1H, *J* = 8.4 Hz), 6.68 (br, s, 1H), 3.36 (br, t, 2H), 1.77 (m, 2H, *J* = 7.2 Hz), 1.03 (t, 3H, *J* = 7.2 Hz). HRMS (ESI+) m/z: calcd for $C_{19}H_{18}N$ $[M+H]^+$ 260.1433; found 260.1434.

Synthesis of **4**. solution of 1-aminopyrene (1001.8 mg, 4.61 mmol) and triethylamine (564.2 mg, 5.58 mmol) in THF (2 mL) was cooled to 0 ℃, and di-*tert*-butyl dicarbonate (997.8 mg, 4.57 mmol) in THF 4 mL was added. The mixture was stirred for 10 min and warmed to room temperature for 2 h. After the removal of the solvent, the residue was purified by silica gel column chromatography (*n*-hexane/ethyl acetate 4:1) to give **4** (1223.2 mg, 3.85 mmol, 84%) as pale orange powder. Compound **4**: m.p. 130–131 ℃; 1H NMR (600 MHz, DMSO-d_6) δ 9.91 (br s, 1H), 8.29–8.24 (m, 4H), 8.18–8.10

(m, 4H), 8.05 (t, 1H, J = 7.8 Hz), 1.54 (s, 9H); ^{13}C NMR (150 MHz, $CDCl_3$) δ 154.7, 133.3, 132.3, 131.8, 128.8, 128.1, 127.7, 126.99, 126.98, 125.82, 125.78, 125.76, 125.42, 125.37, 124.0, 122.6, 122.5, 80.1, 28.4; HRMS (ESI−) m/z: calcd for $C_{21}H_{18}NO_2$ $[M-H]^-$ 316.1343; found 316.1351.

Synthesis of **5**. A solution of **4** (1201.1 mg, 3.78 mmol) and diethylene glychol 2-bromoethyl methyl ether (953.4 mg, 4.20 mmol) in DMF (6.0 mL) was stirred at room temperature for 10 min. Sodium hydride (60%, 169.7 mg, 4.24 mmol) in DMF (5.0 mL), which was washed with *n*-hexane twice, was added to the reaction mixture at 0 ℃, and the mixture was stirred for 3.5 h at 60 ℃. After removal of the solvent, the residue was extracted with dichloromethane and water. The organic layer was dried over magnesium sulfate, filtered and evaporated. The residue was purified by silica gel column chromatography (*n*-hexane/ethyl acetate 4:1) to give **5** (1796.9 mg, 3.88 mmol, quant.) as yellow oil. Compound **5**: ^{1}H NMR (600 MHz, $CDCl_3$) δ 8.20–8.00 (m, 8H), 7.88 (d, 1H, J = 8.4 Hz), 4.16–4.14 (m, 1H), 3.97–3.95 (m, 1H), 3.69–3.62 (m, 3H), 3.56–3.49 (m, 6H), 3.43–3.41 (m, 2H), 3.31 (br, s, 3H), 1.20 (s, 9H); ^{13}C NMR (150 MHz, acetone-d_6) δ 155.7, 137.6, 132.1, 131.9, 131.2, 129.1, 128.6, 128.3, 128.1, 127.4, 127.2, 126.3, 126.2, 126.1, 125.9, 125.4, 123.9, 80.0, 72.6, 71.1, 71.0, 70.9, 69.1, 58.8, 50.6, 28.3; HRMS (ESI−) m/z: calcd for $C_{28}H_{34}NO_5$ $[M-H]^-$ 464.2431; found 464.2436.

Synthesis of **3c**. Trifluoroacetic acid (4494.3 mg, 39.4 mmol) in dry dichloromethane (2.0 mL) was added to a solution of **5** (1777.9 mg, 3.84 mmol) in dichloromethane (2.0 mL), and the mixture was stirred for 1.5 h at room temperature. The reaction mixture was neutralized with saturated aqueous sodium bicarbonate (pH>7), and extracted with dichloromethane. The organic layer was washed with brine, dried over magnesium sulfate, and filtered. The solvent was removed to give **3c** (1235.3 mg, 3.40 mmol, 86%) as orange oil. Compound **3c**: ^{1}H NMR (600 MHz, $CDCl_3$) δ 8.07–7.99 (m, 5H), 7.90 (dd, 2H, J = 7.8, 9.0 Hz), 7.79 (d, 1H, J = 8.4 Hz), 7.41 (br, s, 1H), 3.94 (t, 2H, J = 4.8 Hz), 3.78–3.73 (m, 4H), 3.70–3.68 (m, 2H), 3.65 (t, 2H, J = 4.8 Hz), 3.55–3.54 (m, 2H), 3.31 (br, s, 3H); ^{13}C NMR (150 MHz, acetone-d_6) δ 144.3, 133.5, 132.8, 128.7, 127.5, 126.9, 126.8, 126.5, 126.0, 124.2, 123.7, 123.4, 123.2, 121.4, 117.1, 109.6, 72.7, 71.2, 71.09, 71.07, 70.0, 58.8, 44.4; HRMS (ESI+) m/z: calcd for $C_{23}H_{26}NO_3$ $[M+H]^+$ 364.1907; found 364.1908.

Synthesis of **6a**. A solution of **3a** (264.2 mg, 1.14 mmol) in ethanol (6.0 mL) was added to a mixture of 3,4-diethoxy-3 cyclobutene-1,2-dione (204.8 mg, 1.20 mmol) and zinc trifluoromethanesulfonate (84.4 mg, 0.23 mmol) in ethanol (2.0 mL), and the mixture was heated at reflux for 21 h under Ar atmosphere. After removal of the solvent, the residue was extracted with dichloromethane and water. The organic layer was dried over magnesium sulfate, filtered and evaporated. The residue was purified by silica gel column chromatography (*n*-hexane/ethyl acetate 2:1) to give **6a** (226.9 mg, 0.64 mmol, 56%) as green powder. Compound Ⅳ-8a: m.p. 179 ℃; ^{1}H NMR (600 MHz, DMSO-d_6) δ 8.38–8.12 (m, 9H), 4.13 (br, q, 2H, J = 6.0 Hz), 3.90 (s, 3H) , 0.71 (br, t, 3H, J = 6.0 Hz); ^{13}C NMR (150 MHz, $CDCl_3$) δ 189.0, 184.2, 178.6, 172.9, 135.5, 131.6, 131.4, 131.2, 130.7, 129.3, 128.5, 127.1, 126.8, 126.3, 126.1, 125.2, 125.0, 124.6, 123.7, 120.8, 69.3, 41.5, 15.3; HRMS (ESI+) m/z: calcd for $C_{23}H_{18}NO_3$ $[M+H]^+$ 356.1281; found 356.1283.

Synthesis of **6b**. A solution of **3b** (128.0 mg, 0.494 mmol) in ethanol (4.0 mL) was added to a mixture of 3,4-diethoxy-3 cyclobutene-1,2-dione (83.9 mg, 0.493 mmol) and zinc trifluoromethanesulfonate (38.5 mg, 0.106 mmol) in ethanol (1.0 mL), and the mixture was heated at reflux for 24 h under Ar atmosphere. Zinc trifluoromethanesulfonate (33.9 mg, 0.093 mmol) was added to the mixture, and the mixture was heated at reflux for 6 h, then cooled to room temperature. After removal of the solvent, the residue was extracted with ethyl acetate and water. The organic layer was washed

with brine, dried over sodium sulfate, filtered and evaporated. The residue was purified by silica gel column chromatography (*n*-hexane/ethyl acetate 2:1), and the mixture was purified by silica gel column chromatography (*n*-hexane/ethyl acetate 3:1) to give **6b** (113.5 mg, 0.296 mmol, 60%) as light green powder. Compound **6b**: m.p. 156–159 ℃; ^{1}H NMR (600 MHz, $CDCl_3$) δ 8.27 (t, *J* = 7.2 Hz, 2H), 8.20–8.16 (m, 3H), 8.12–8.07 (m, 2H), 8.00 (d, *J* = 9.0 Hz, 1H), 7.87 (d, *J* = 7.8 Hz, 1H), 4.94 (m, 0.30×2H), 4.44–4.39 (m, 0.70×2H), 4.27–4.19 (m, 2H), 1.72–1.66 (m, 2H), 1.59 (t, *J* = 7.2 Hz, 0.30×3H), 0.97 (t, *J* = 7.2 Hz, 3H), 0.78 (t, *J* = 7.2 Hz, 0.70×3H); 13C NMR (150 MHz, CDCl3) δ 188.6, 184.5, 178.3, 172.9, 133.6, 131.3, 131.2, 130.7, 129.2, 128.5, 127.5, 127.1, 126.7, 126.3, 126.04, 125.2, 124.8, 124.6, 124.5, 121.2, 69.2, 55.5, 22.3, 15.3, 11.0. HRMS (ESI+) m/z: calcd for $C_{25}H_{21}NNaO_3$ $[M+Na]^+$ 406.1414; found 406.1424.

Synthesis of **6c**. A solution of **3c** (870.5 mg, 2.400 mmol) in ethanol (6.0 mL) was added to a mixture of 3,4-diethoxy-3 cyclobutene-1,2-dione (414.9 mg, 2.438 mmol) and zinc trifluoromethanesulfonate (175.6 mg, 0.483 mmol) in ethanol (4.0 mL), and the mixture was heated at reflux for 1 day under Ar atmosphere. After removal of the solvent, the residue was extracted with dichloromethane and water. The organic layer was dried over magnesium sulfate, filtered and evaporated. The residue was purified by silica gel column chromatography (*n*-hexane/ethyl acetate 1:1) to give **6c** (1.0435 g, 2.14 mmol, 89%) as yellow green oil. Compound **6c**: ^{1}H NMR (600 MHz, $CDCl_3$, 313 K, major : minor = 74 : 26) δ 8.27–8.06 (m, 8H), 7.98 (d, 1H, *J* = 8.4 Hz), 4.93 (br, s, minor 0.26×2H), 4.60 (m, 1H), 4.50 (m, 1H), 4.24–4.20 (m, 2H), 3.62 (q, major 0.74×2H, *J* = 6.6 Hz), 3.61–3.57 (m, 6H), 3.47 (m, 2H), 3.34 (s, 3H), 1.59 (br, s, minor 0.26×3H), 0.76 (t, major 0.74×3H, *J* = 6.6 Hz); ^{13}C NMR (150 MHz, acetone-d_6) δ 188.5, 184.0, 177.7, 173.4, 134.0, 131.23, 131.19, 130.9, 128.7, 128.2, 128.0, 127.2, 126.7, 125.9, 125.8, 125.5, 125.0, 124.7, 124.3, 122.2, 71.8, 70.29, 70.27, 70.2, 68.4, 67.7, 57.9, 52.7, 14.6; HRMS (ESI+) m/z: calcd for $C_{29}H_{29}NNaO_6$ $[M+Na]^+$ 510.1887; found 510.1892.

Synthesis of **1a**. *p*-Toluidine (124.1 mg, 1.158 mmol) was added to a mixture of **6a** (186.2 mg, 0.524 mmol) and zinc trifluoromethanesulfonate (39.2 mg, 0.108 mmol) in toluene/DMF 19:1 (8.6 mL), and the mixture was heated at reflux for 1 day under Ar atmosphere. After removal of the solvent, the residue was extracted with dichloromethane and 2N HCl. The organic layer was dried over magnesium sulfate, filtered and evaporated. The residue was purified by silica gel column chromatography (*n*-hexane/ethyl acetate 2:1) to give **1a** (108.8 mg, 0.261 mmol, 50%) as yellow amorphous. Compound **1a**: ^{1}H NMR (600 MHz, $CDCl_3$) δ 8.33 (d, 1H, *J* = 7.2 Hz), 8.31 (d, 1H, *J* = 7.8 Hz), 8.27 (d, 2H, *J* = 9.0 Hz), 8.23 (d, 1H, *J* = 9.0 Hz), 8.15–8.12 (m, 3H), 8.01 (d, 1H, *J* = 8.4 Hz), 6.73 (d, 2H, *J* = 7.8 Hz), 6.46 (d, 2H, *J* = 9.0 Hz), 5.03 (br, s, 1H), 4.07 (s, 3H), 2.03 (s, 3H); ^{13}C NMR (150 MHz, $CDCl_3$) δ 184.7, 181.9, 167.8, 165.2, 134.7, 134.0, 133.6, 131.8, 131.2, 130.8, 130.5, 129.5, 129.3, 127.3, 127.1, 127.0, 126.8, 126.7, 125.2, 124.4, 124.3, 120.6, 118.9, 41.9, 20.6; HRMS (ESI+) m/z: calcd for $C_{28}H_{21}N_2O_2$ $[M+H]^+$ 417.1600; found 417.1608.

Synthesis of **1b**. *p*-Toluidine (31.2 mg, 0.291 mmol) was added to a mixture of **6b** (104.1 mg, 0.271 mmol) and zinc trifluoromethanesulfonate (20.8 mg, 0.057 mmol) in toluene/DMF 19:1 (5.0 mL), and the mixture was heated at reflux for 16 h under Ar atmosphere. A solusion of *p*-toluidine (30.6 mg, 0.286 mmol) was added to the mixture, and the mixture was heated at reflux for 8 h, then cooled to room temperature. After removal of the solvent, the residue was extracted with dichloromethane and 2N HCl. The organic layer was washed with brine, dried over sodium sulfate, filtered and evaporated. The residue was purified by silica gel column chromatography (*n*-hexane/ethyl acetate 2:1) to give **1b** (39.6 mg, 0.089 mmol, 33%) as yellow solid. Compound **1b**: m.p. 186–187 ℃; ^{1}H NMR (600 MHz, $CDCl_3$) δ 8.33 (d, *J* = 7.2 Hz, 1H), 8.31 (d, *J* = 7.8 Hz, 1H), 8.28–8.23 (m, 3H), 8.15 (q, *J* = 8.4 Hz, 3H), 8.00 (d, *J* = 7.8 Hz, 1H), 6.74 (d, *J* =

7.8 Hz, 2H), 6.46 (d, J = 9.0 Hz, 2H), 4.97 (br, s, 1H), 4.61–4.58 (m, 1H), 4.26–4.22 (m, 1H), 2.05 (s, 3H), 1.78–1.72 (m, 2H), 1.01 (t, J = 7.2 Hz, 3H); ^{13}C NMR (150 MHz, $CDCl_3$) δ 184.3, 181.8, 167.7, 164.8, 134.8, 133.5, 132.2, 131.8, 131.3, 130.8, 130.4, 129.5, 129.3, 127.8, 127.3, 127.0, 126.8, 126.6, 125.3, 125.2, 124.9, 124.4, 120.9, 118.8, 55.7, 22.6, 20.6, 11.0; HRMS (ESI+) m/z: calcd for $C_{30}H_{24}N_2NaO_2$ $[M+Na]^+$ 467.1730; found 467.1735.

Synthesis of **1c**. *p*-Toluidine (45.1 mg, 0.429 mmol) was added to a mixture of **6c** (197.2 mg, 0.404 mmol) and zinc trifluoromethanesulfonate (29.8 mg, 0.082 mmol) in toluene/DMF 19:1 (6.0 mL), and the mixture was heated at reflux for 1 day under Ar atmosphere. After removal of the solvent, the residue was extracted with dichloromethane and 2N HCl. The organic layer was dried over magnesium sulfate, filtered and evaporated. The residue was purified by silica gel flash column chromatography (*n*-hexane/ethyl acetate 1:2) to give **1c** (125.0 mg, 0.228 mmol, 56%) as yellow green oil. Compound **1c**: ^{1}H NMR (600 MHz, $CDCl_3$) δ 8.33–8.13 (m, 8H), 6.75 (br, s, 2H), 6.46 (br, s, 2H), 4.96 (br, s, 1H), 4.81 (br, s, 1H), 4.44 (d, 1H, J = 13.2 Hz), 3.69–3.58 (m, 8H), 3.46 (t, 2H, J = 4.2 Hz), 3.30 (s, 3H), 2.06 (br, s, 3H); ^{13}C NMR (150 MHz, $CDCl_3$) δ 184.2, 181.8, 168.0, 164.7, 134.8, 133.4, 132.0, 131.8, 131.2, 130.8, 130.4, 129.5, 129.3, 128.0, 127.2, 127.0, 126.7, 126.6, 125.9, 125.0, 124.3, 121.0, 118.6, 72.0, 70.7, 70.6, 70.5, 68.4, 59.1, 53.2, 20.6; HRMS (ESI−) m/z: calcd for $C_{34}H_{31}N_2O_5$ $[M-H]^-$ 547.2238; found 547.2260.

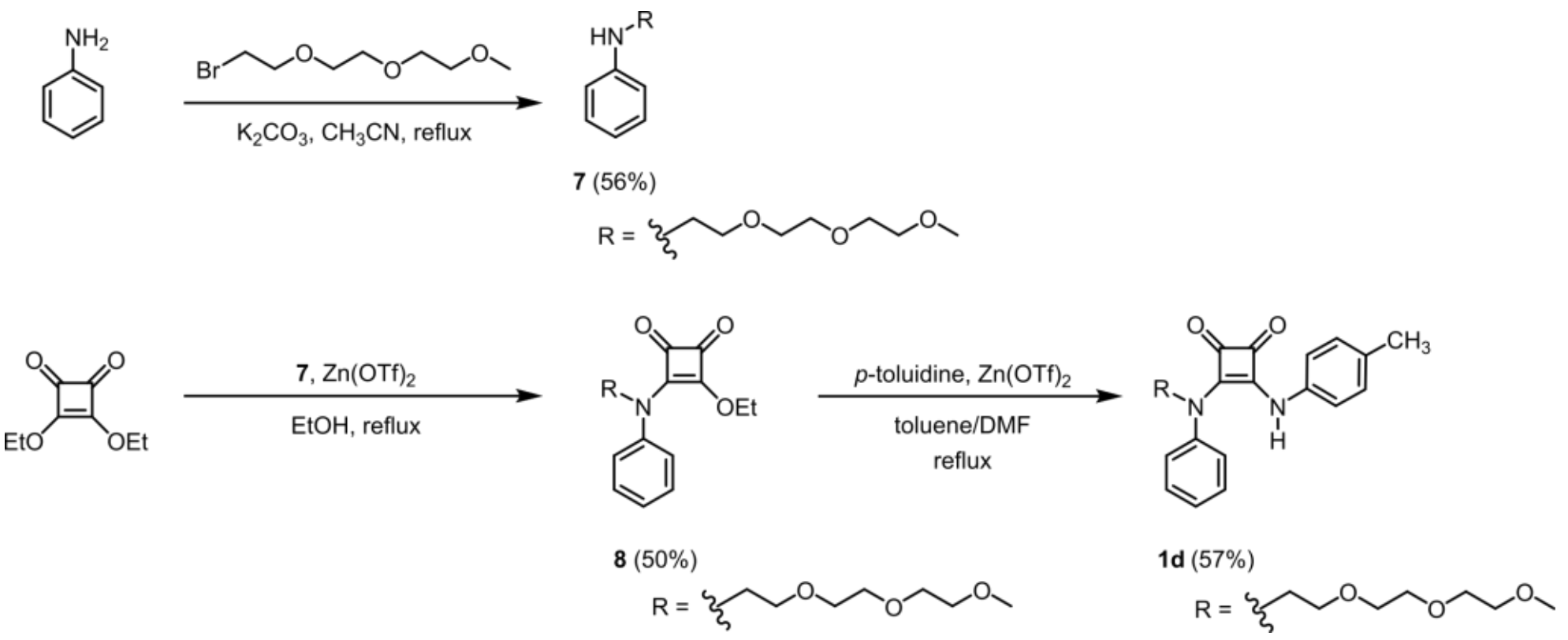

Scheme S2. Synthetic scheme of compound **1d**

Synthesis of **7**. A solution of diethylene glycol 2-bromoethyl methyl ether (1.2259 g, 5.398 mmol) in dry acetonitrile (4 mL) was added to a solution of aniline (510.8 mg, 5.485 mmol) in dry acetonitrile (4 mL), and the mixture was stirred. Potassium carbonate (3.7209 g, 26.92 mmol) was added to the mixture, and the mixture was stirred at 80 ℃ for 29 h, then cooled to room temperature. The reaction mixture was evaporated and extracted with chloroform and water. The organic layer was washed with brine, dried over sodium sulfate, filtered and evaporated. The residue was purified by silica gel column chromatography (*n*-hexane/ethyl acetate 7:3) to give **7** as a yellow oil (725.2 mg, 3.030 mmol, 56%). Compound **7**: ^{1}H NMR (600 MHz, $CDCl_3$) δ 7.17 (dd, J = 5.4, 7.8 Hz, 2H), 6.70 (tt, J = 1.2, 7.2 Hz, 1H), 6.63 (dd, J = 1.2, 7.2 Hz, 2H), 3.70 (t, J = 5.4 Hz, 2H), 3.66–3.64 (m, 6H), 3.56–3.54 (m, 2H), 3.38 (s, 3H), 3.29 (t, J = 5.4 Hz, 2H); ^{13}C NMR (125 MHz, $CDCl_3$) δ 148.1, 129.3, 117.8, 113.4, 72.0, 70.7, 70.4, 70.3, 69.6, 59.2, 43.8; HRMS (ESI+) m/z: calcd for $C_{13}H_{21}NNaO_3$ $[M+Na]^+$ 262.1414; found 262.1418.

Synthesis of **8**. A solution of 3,4-diethoxy-3-cyclobutene-1,2-dione (352.4 mg, 2.071 mmol) and zinc trifluoromethanesulfonate (149.3 mg, 0.411 mmol) in dry ethanol (8 mL) was stirred at room temperature for 10 min.

A solution of **7** (536.4 mg, 2.241 mmol) in dry ethanol (5 mL) was added to the mixture, and the mixture was heated at reflux for 29 h, then cooled to room temperature. The reaction mixture was evaporated and extracted with dichloromethane and water. The organic layer was washed with brine, dried over sodium sulfate, filtered and evaporated. The residue was purified by silica gel column chromatography (*n*-hexane/ethyl acetate 1:3) to give **8** as a yellow oil (377.0 mg, 1.037 mmol, 50%). Compound **8**: ^{1}H NMR (600 MHz, $CDCl_3$) δ 7.38 (t, *J* = 7.8 Hz, 2H), 7.31 (tt, *J* = 1.2, 7.2 Hz, 1H), 7.24 (d, *J* = 7.2 Hz, 2H), 4.64 (br, s, 0.51×2H), 4.29 (br, s, 0.49×2H), 3.62 (br, t, *J* = 5.4 Hz, 2H), 3.60–3.57 (m, 6H), 3.51 (m, 2H), 3.36 (s, 3H), 1.28 (br, s, 3H); ^{13}C NMR (150 MHz, $CDCl_3$) δ 188.8, 184.4, 177.5, 171.4, 140.0, 129.0, 127.6, 125.3, 72.0, 70.7, 70.5, 69.7, 68.5, 59.1, 52.6, 51.9, 15.8; HRMS (ESI+) m/z: calcd for $C_{19}H_{25}NNaO_6$ $[M+Na]^+$ 386.1574; found 386.1576.

Synthesis of **1d**. A solution of **8** (111.8 mg, 0.308 mmol) and zinc trifluoromethane sulfonate (24.5 mg, 0.067 mmol) in toluene/DMF 19:1 (4 mL) was stirred at room temperature for 10 min. A solution of *p*-toluidine (34.7 mg, 0.324 mmol) in toluene (1 mL) was added to the mixture, and the mixture was heated at reflux for 22.5 h. A solution of *p*-toluidine (20.2 mg, 0.189 mmol) was added to the mixture, and the mixture was heated at reflux for 3.5 h, then cooled to room temperature. The reaction mixture was evaporated and extracted with dichloromethane and water. The organic layer was washed with brine, dried over sodium sulfate, filtered and evaporated. The residue was purified by silica gel column chromatography (*n*-hexane/ethyl acetate 1:2) to give **1d** as a brown oil (74.1 mg, 0.175 mmol, 57%). Compound **1d**: ^{1}H NMR (600 MHz, $CDCl_3$) δ 7.49 (tt, *J* = 2.4, 7.8 Hz, 2H), 7.40 (t, *J* = 7.2 Hz, 1H), 7.36 (d, *J* = 7.2 Hz, 2H), 7.04 (q, *J* = 8.4 Hz, 4H), 4.31 (br t, *J* = 4.8 Hz, 2H), 3.71 (t, *J* = 4.8 Hz, 2H), 3.66–3.64 (m, 2H), 3.62–3.60 (m, 2H), 3.58 (tt, *J* = 1.8, 4.8 Hz, 2H), 3.48 (tt, *J* = 1.8, 4.8 Hz, 2H), 3.31 (s, 3H), 2.26 (s, 3H); ^{13}C NMR (125 MHz, $CDCl_3$) δ 184.5, 182.5, 166.9, 164.9, 139.9, 135.4, 133.6, 129.9, 128.0, 125.6, 118.9, 72.0, 70.7, 70.5, 68.7, 50.1, 59.1, 52.3, 20.9; HRMS (ESI+) m/z: calcd for $C_{24}H_{28}N_2NaO_5$ $[M+Na]^+$ 447.1890; found 447.1892.

1.3. ^{1}H and ^{13}C{^{1}H} NMR charts of compounds 1a – 1d

^{1}H NMR of **1a**

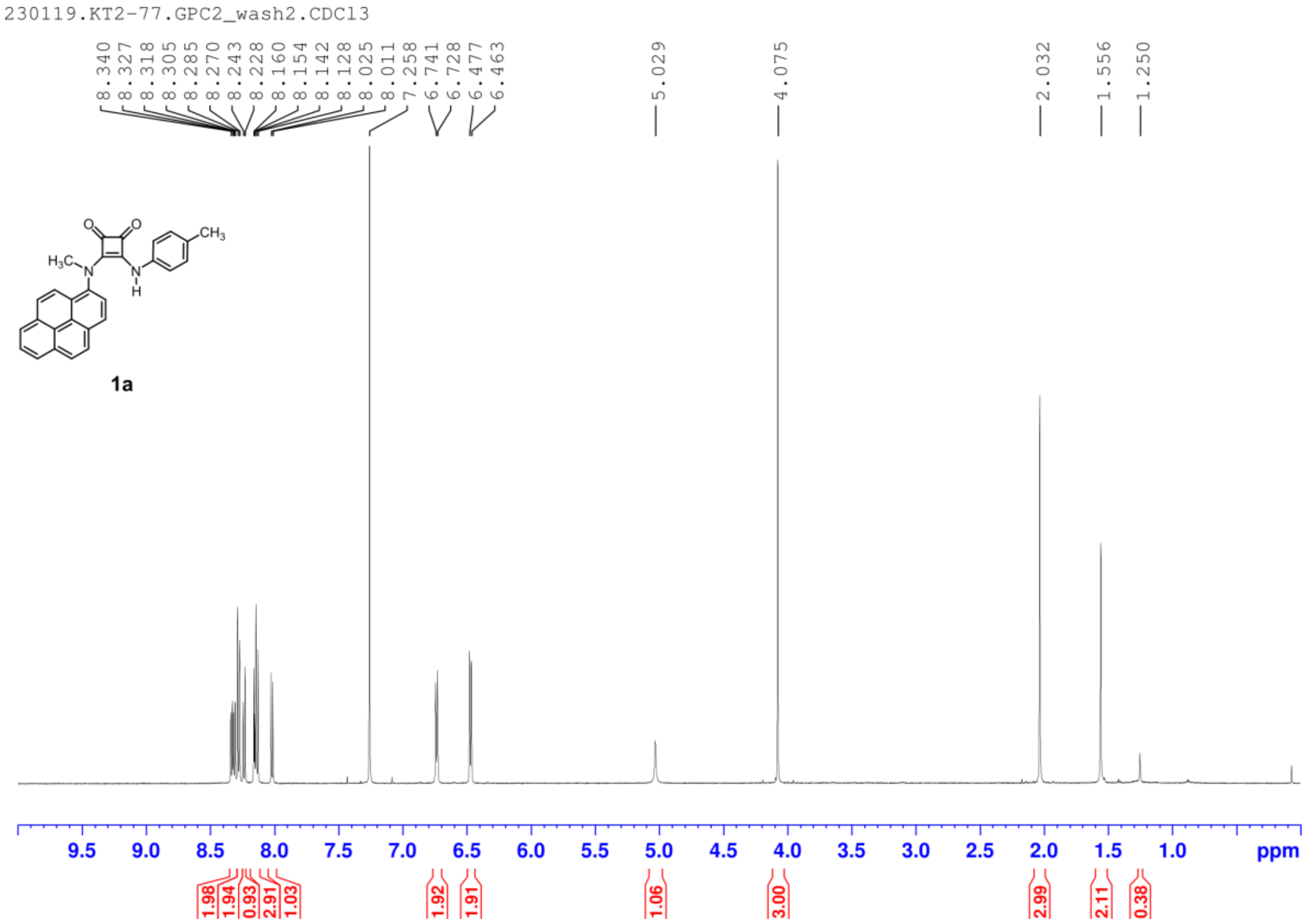

^{13}C{^{1}H} NMR of **1a**

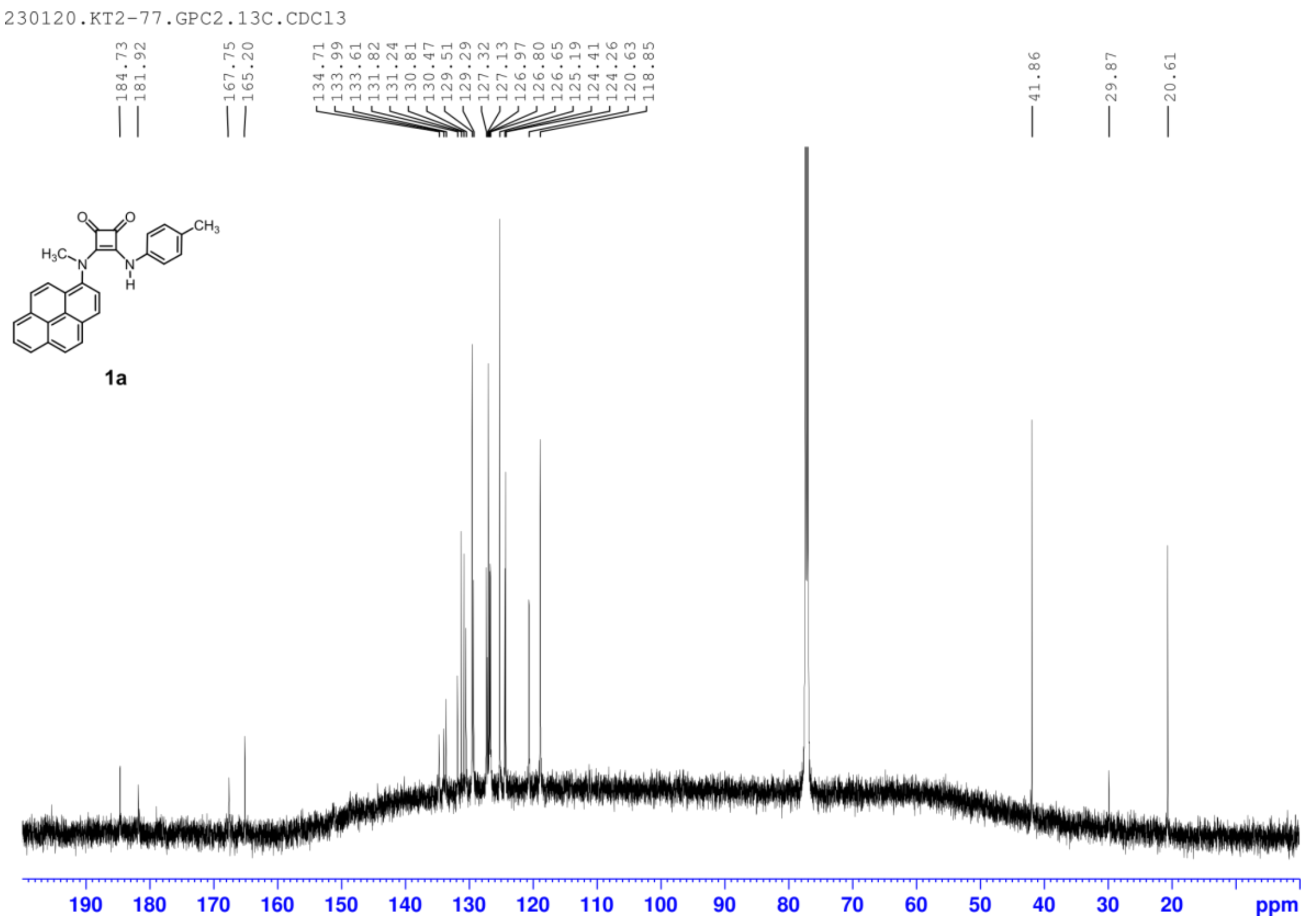

^{1}H NMR of **1b**

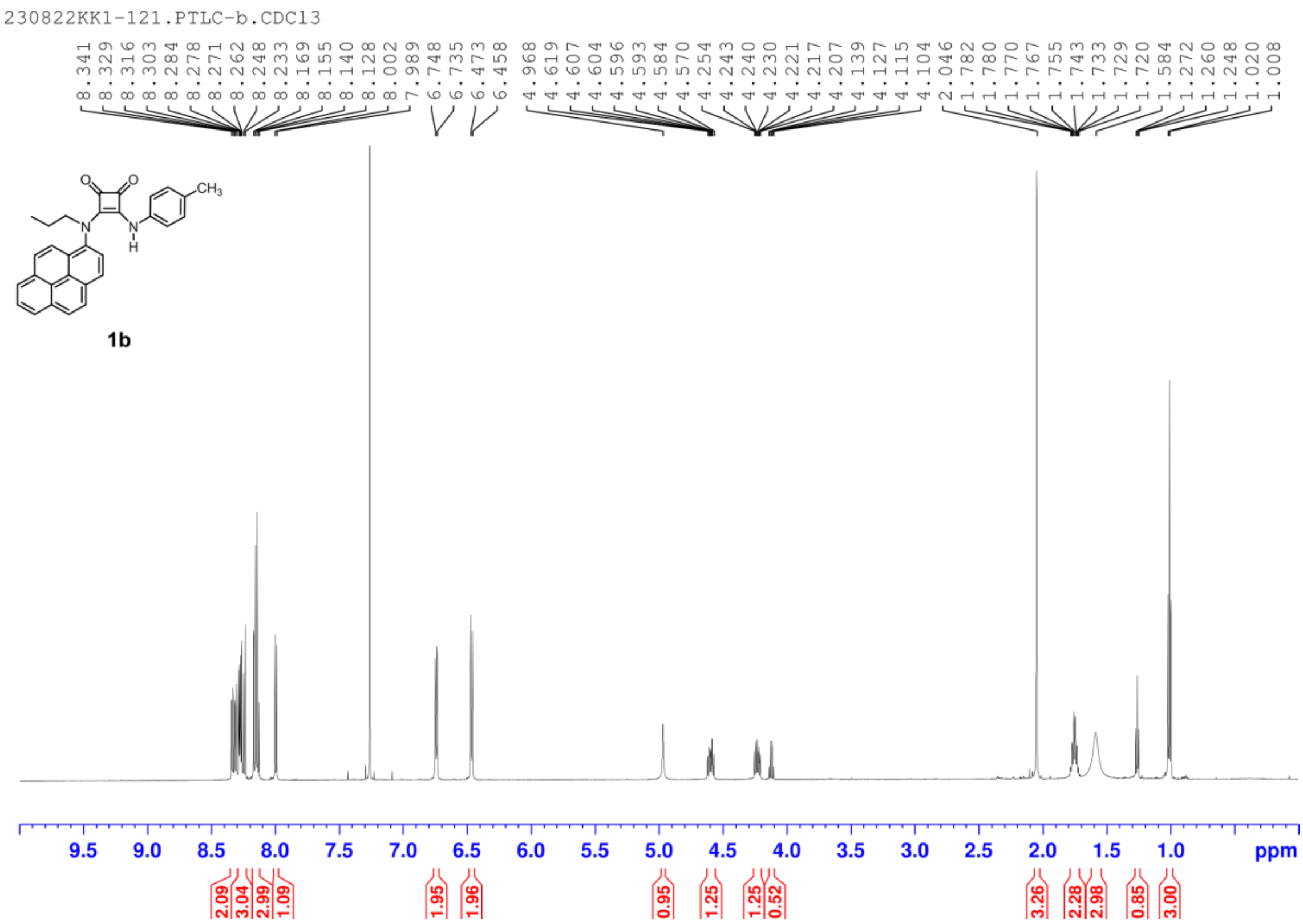

^{13}C{^{1}H} NMR of **1b**

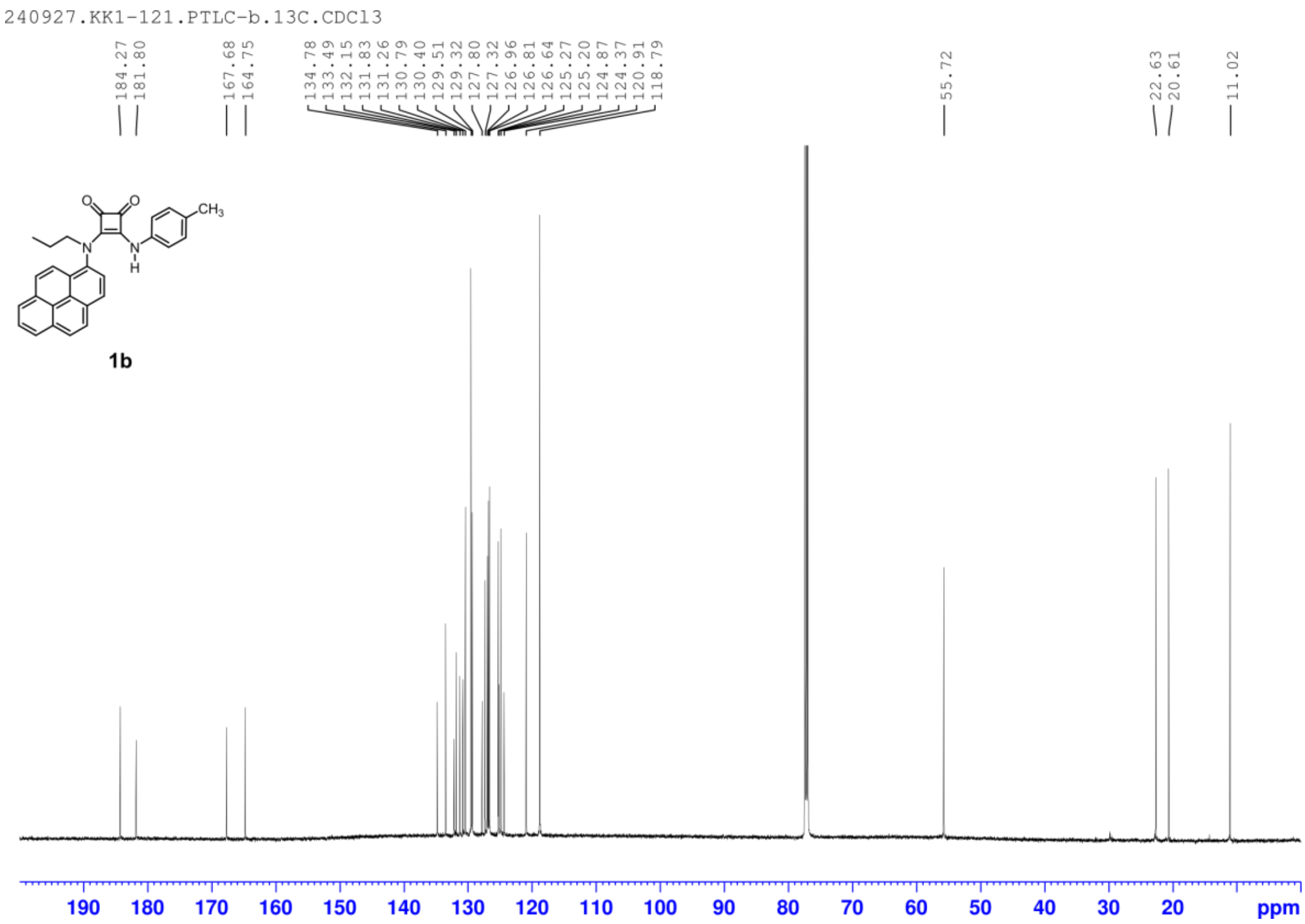

^{1}H NMR of **1c**

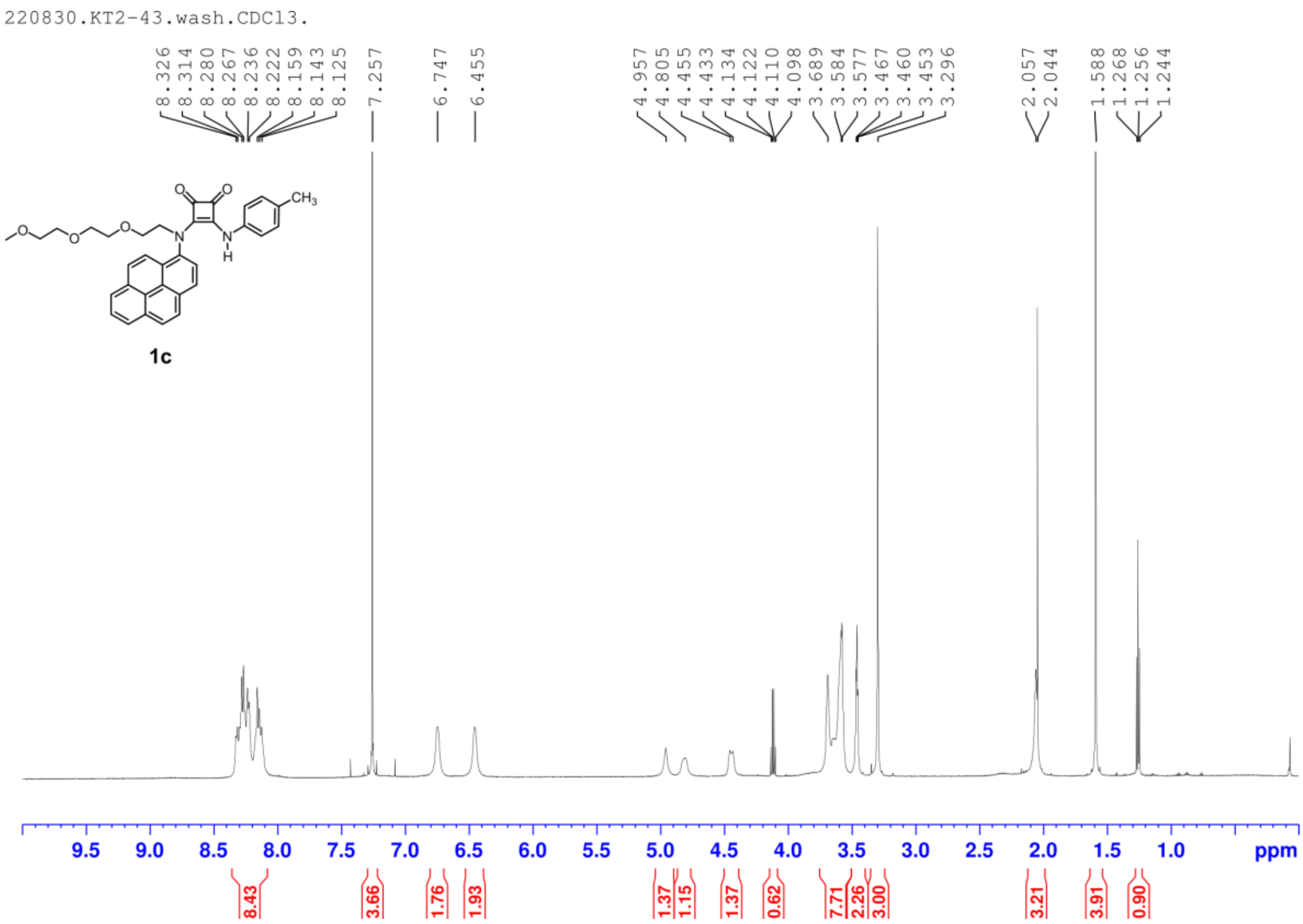

$^{13}C\{^{1}H\}$ NMR of **1c**

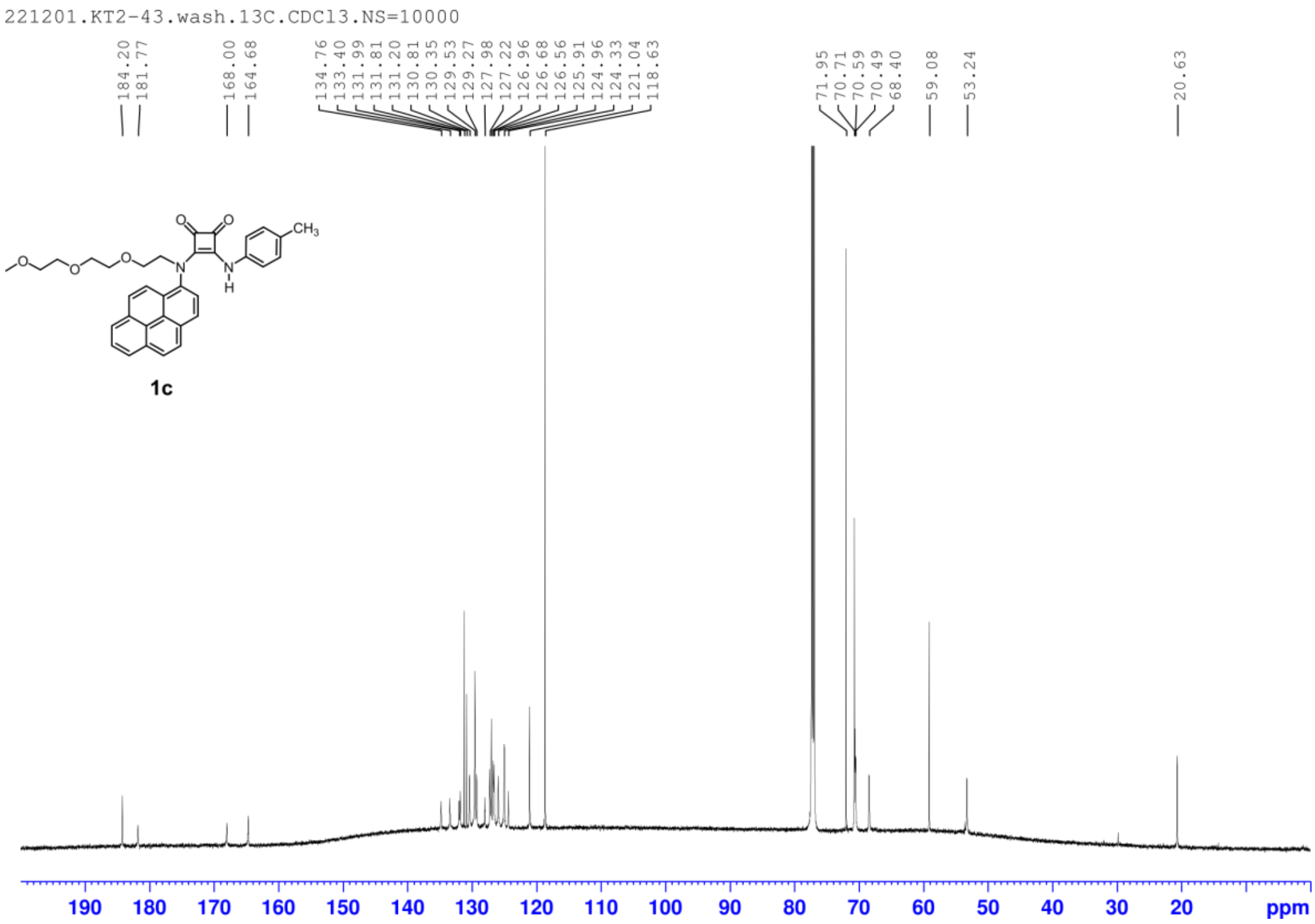

^{1}H NMR of **1d**

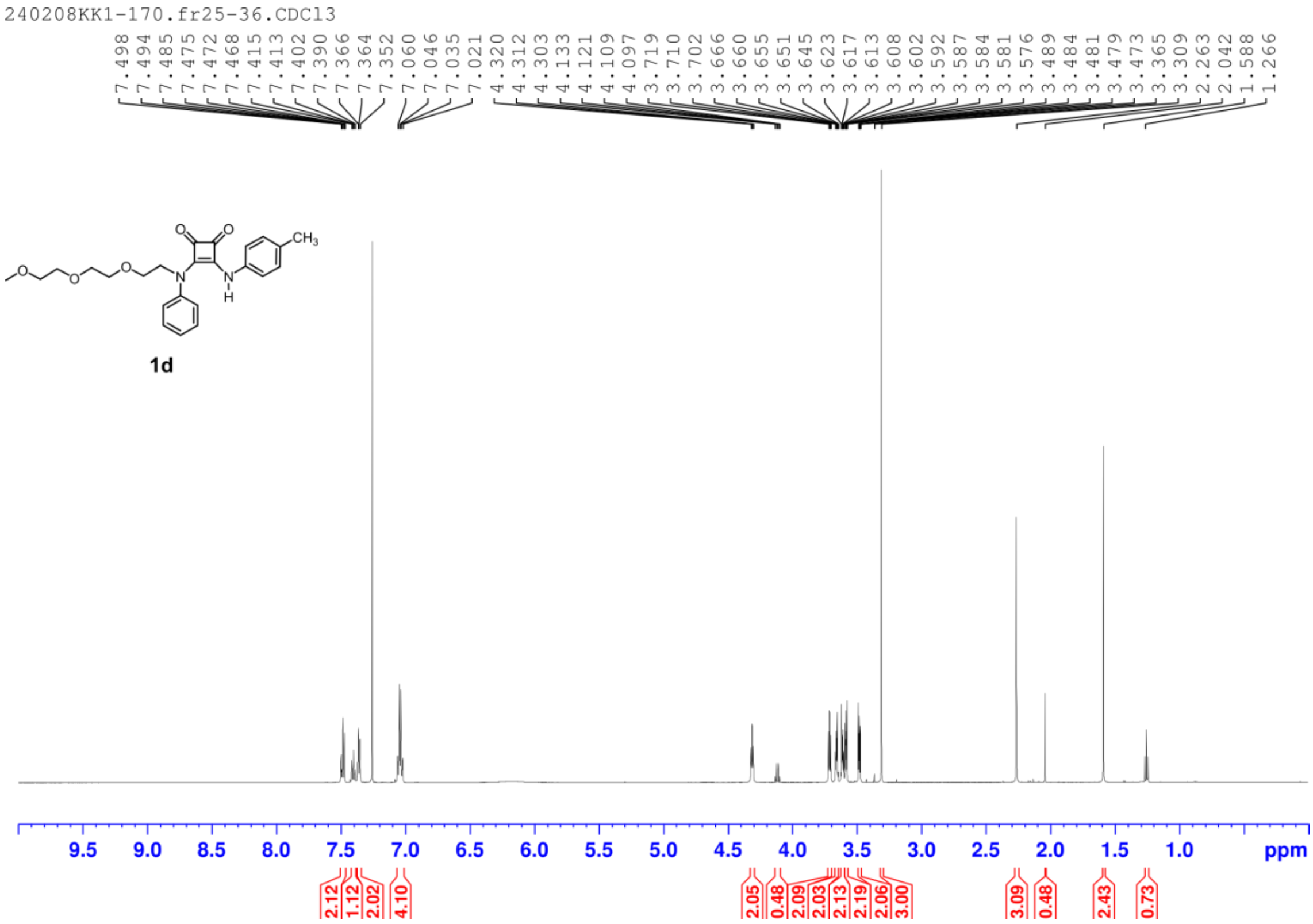

$^{13}C\{^{1}H\}$ NMR of **1d**

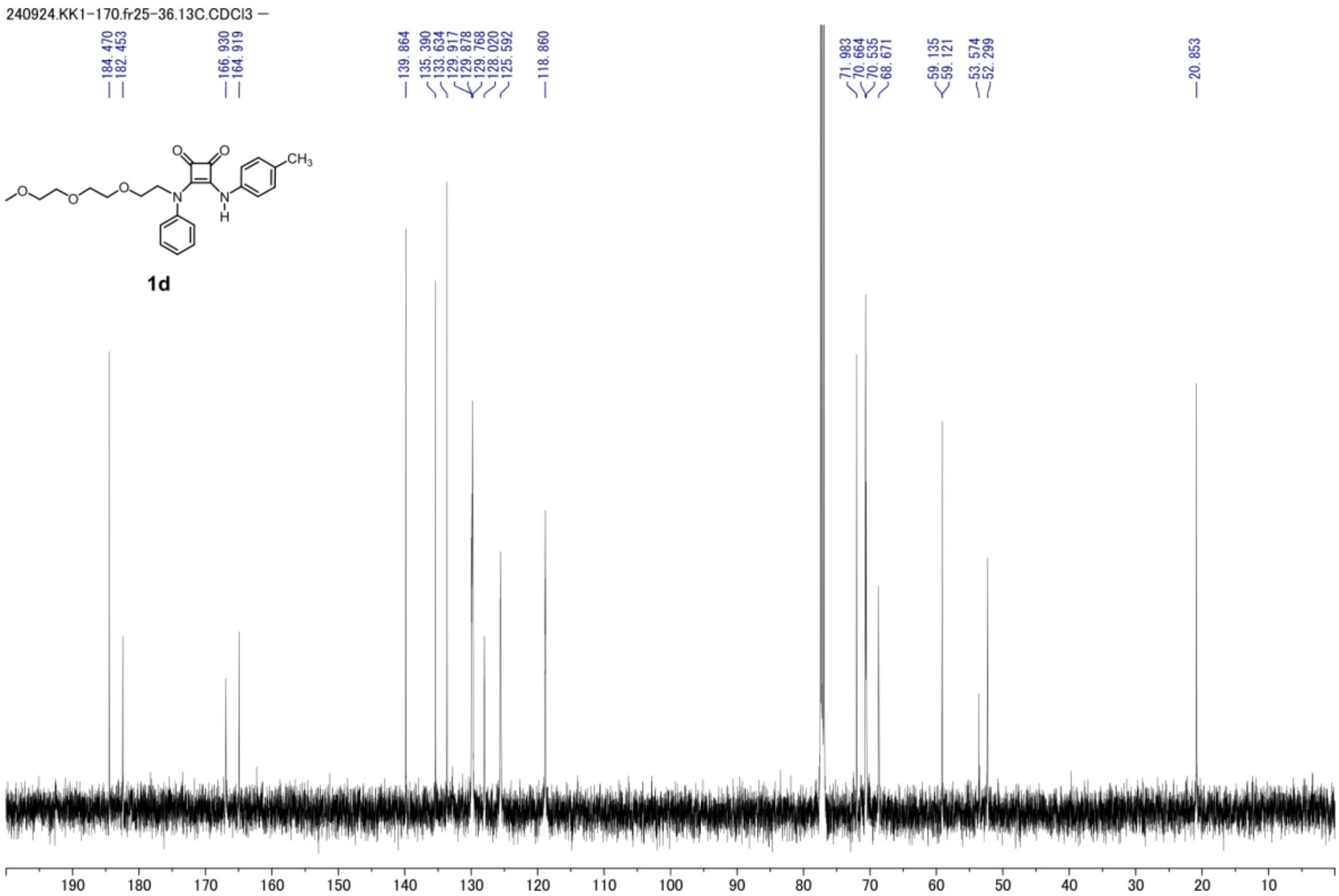

2. X-Ray Crystallographic Analysis of compounds 1a and 1b

A single crystal was immersed in Paratone-N oil and placed in a stream of cold N_2 at 183 K for **1a** or 100 K for **1b**. Diffraction data were collected with a Bruker APEX2 CCD detector (Bruker APEX2 Ultra, Mo Kα: λ = 0.71073 Å) for **1a** or an EIGER 4M PAD detector (SPring-8 BL26B1, synchrotron: λ = 0.800 Å) for **1b**. Structure solution and refinement were performed by using SHELXT-2014/5 or SHELXT-2018/2,[28] and SHELXL-2018/3 or SHELXL-2019/2.[29] CCDC Deposition Number are 2541946 for **1a** and 2541947 for **1b**. These data are provided free of charge by the joint https://www.ccdc.cam.ac.uk/structures/.

Table S1. Crystal data of **1a** and **1b**

		**1a**	**1b**
Recryst. solv.		methanol/chloform	AcOEt/EtOH
Formula		$C_{28}H_{20}N_2O_2$	$C_{30}H_{24}N_2O_2$
Formula weight		416.46	444.51
Temperature (K)		183(2)	100(2)
Crystal system		triclinic	monoclinic
Space group		$P\overline{1}$	C2/*c*
Unit cell dimensions	*a* (Å)	11.3106(12)	24.051(5)
	b (Å)	13.4250(14)	9.2660(19)
	c (Å)	13.8928(14)	20.282(4)
	α (deg)	87.300(2)	90
	β (deg)	88.322(2)	102.95(3)
	γ (deg)	85.633(2)	90
V ($Å^3$)		2100.4 (4)	4405.0(16)
Z value		4	8
ρ_{calc} (g cm^{-3})		1.317	1.341
μ (mm^{-1})		0.084	0.108
F(000)		872.0	1872.0
Crystal size (mm^3)		0.11×0.10 ×0.08	0.05×0.04 ×0.02
Radiation		MoKα (λ = 0.71073)	synchrotron (λ = 0.800)
2Θ range for data collection (°)		2.936 to 63.004	3.912 to 66.276
Index ranges		-16 ≤ h ≤ 10, -15 ≤ k ≤ 18, -20 ≤ l ≤ 20	-32 ≤ h ≤ 32, -11 ≤ k ≤ 11, -24 ≤ l ≤ 24
Independent reflections		12609 [R_{int} = 0.0270, R_{sigma} = 0.0587]	4617 [R_{int} = 0.0414, R_{sigma} = 0.0248]
Goodness-of-fit on F^2		1.019	1.304
Final R indexes [I>=2σ(I)]		R_1 = 0.0645, wR_2 = 0.1633	R_1 = 0.1214, wR_2 = 0.2837
Final R indexes [all data]		R_1 = 0.1005, wR_2 = 0.1826	R_1 = 0.1459, wR_2 = 0.3230
Largest diff. peak/hole (e $Å^{-3}$)		0.39/-0.27	0.92/-0.88
CCDC number		2541946	2541947

3. Crystal Packing structures of compounds **1a** and **1b** (Figure S1)

(a)

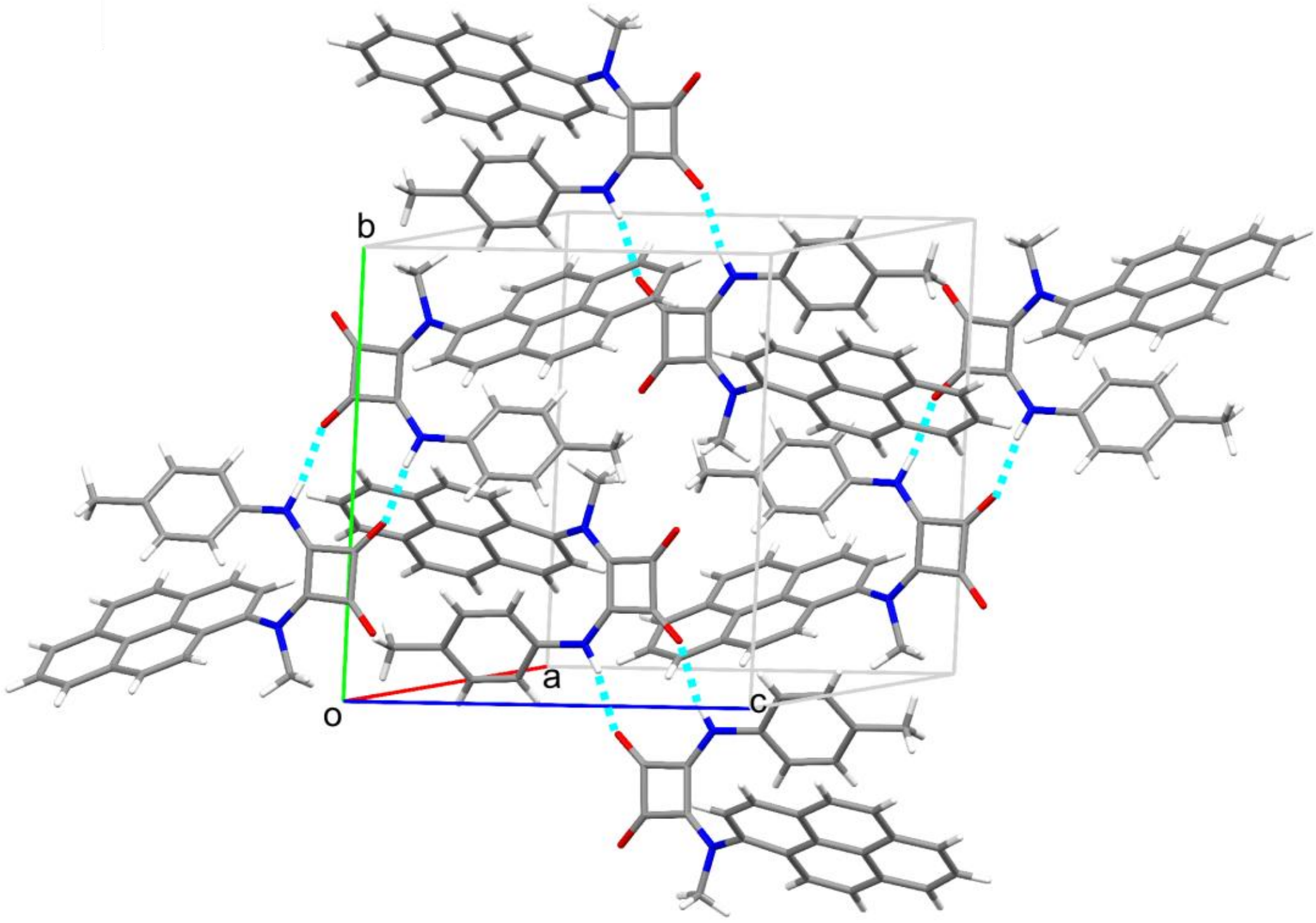

(b)

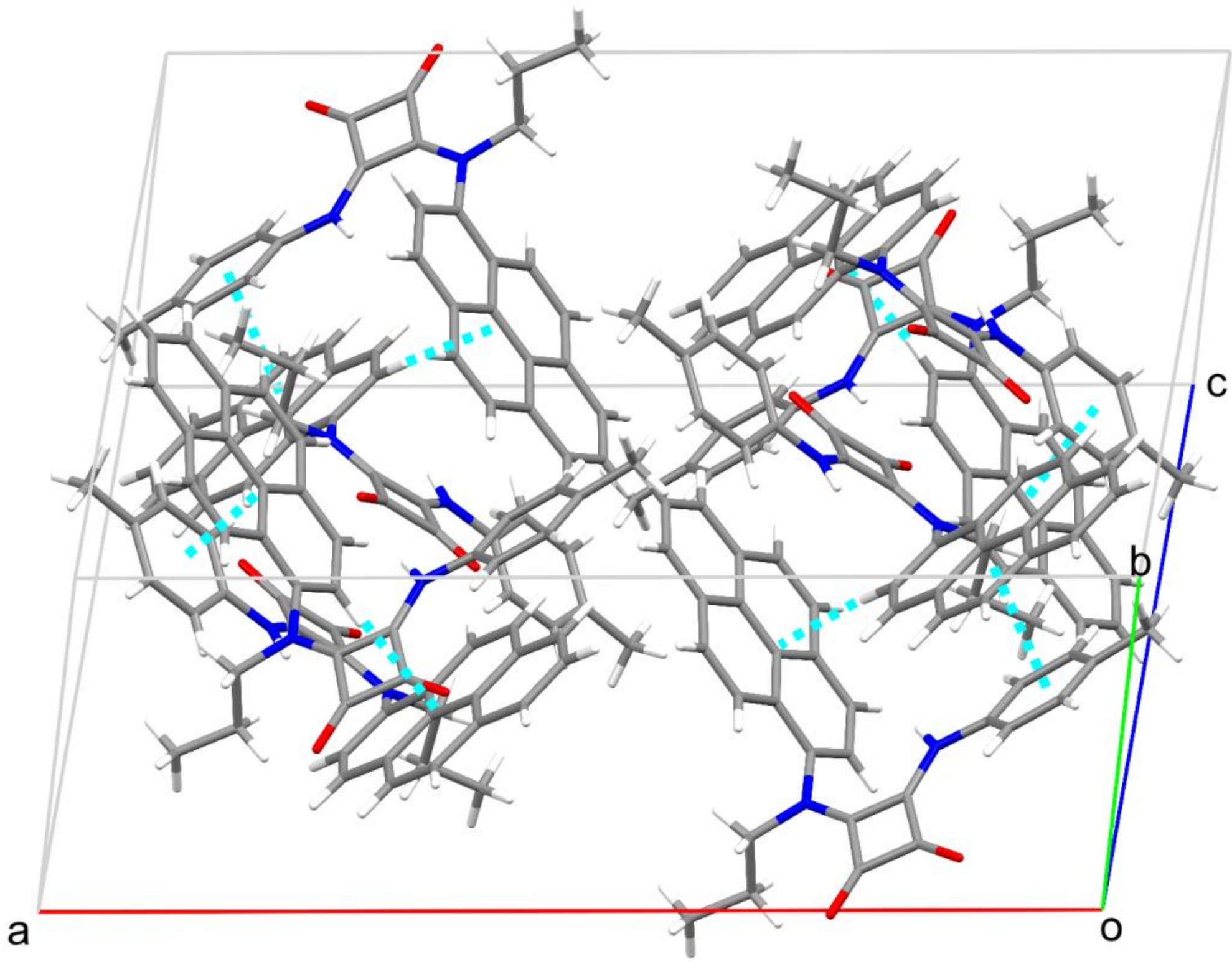

Figure S1. Crystal Packing structures of compounds (a) **1a** and (b) **1b**

4. Temperature-dependent ^{1}H NMR spectra of compounds 1a and 1b

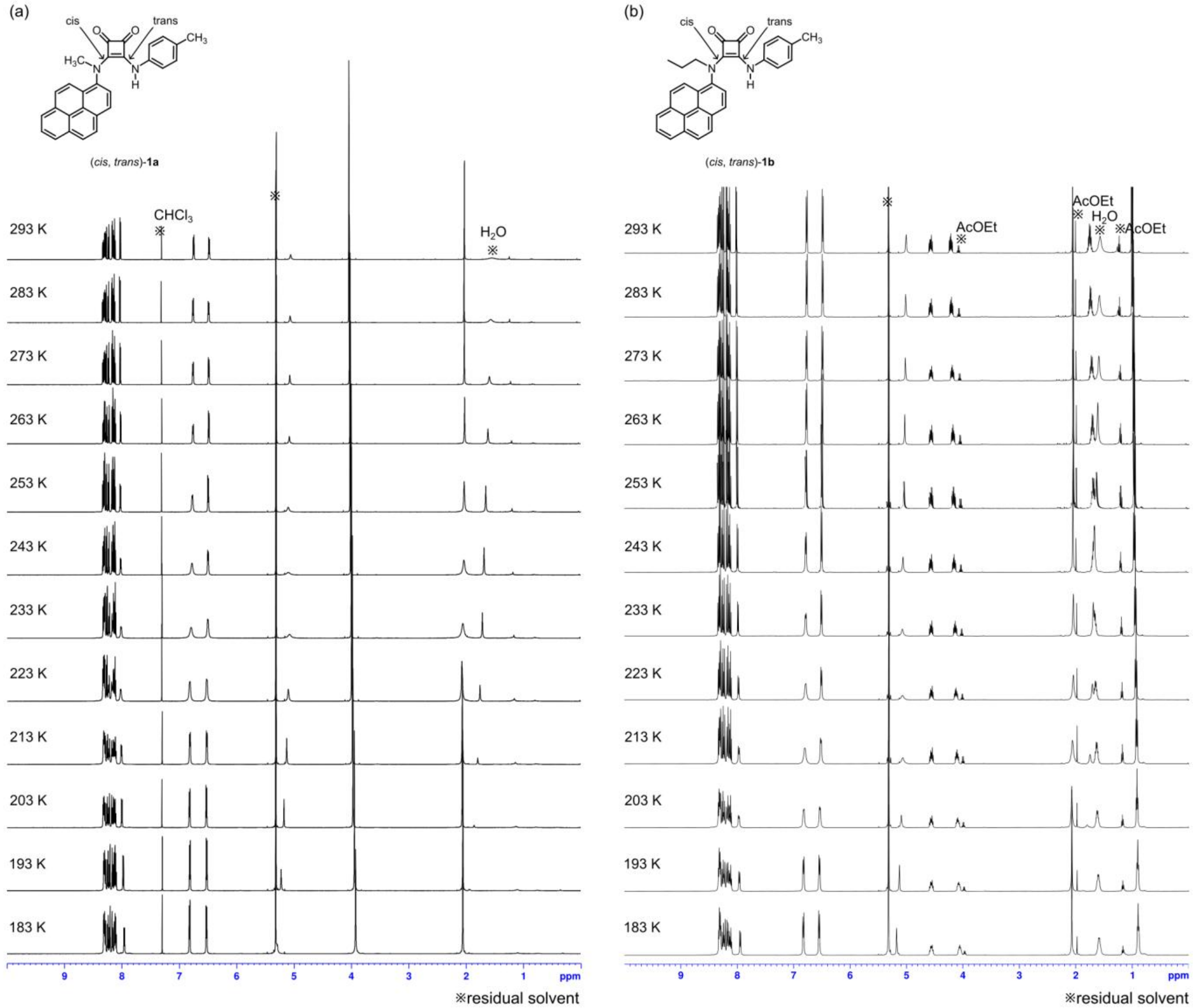

Figure S2. Temperature-dependent ^{1}H NMR spectra of (a) **1a** (600 MHz, 10 mM) and (b) **1b** (500 MHz, 10 mM) in CD_2Cl_2

5. NMR spectra and computational details of compound 1c

5.1. NOESY spectra

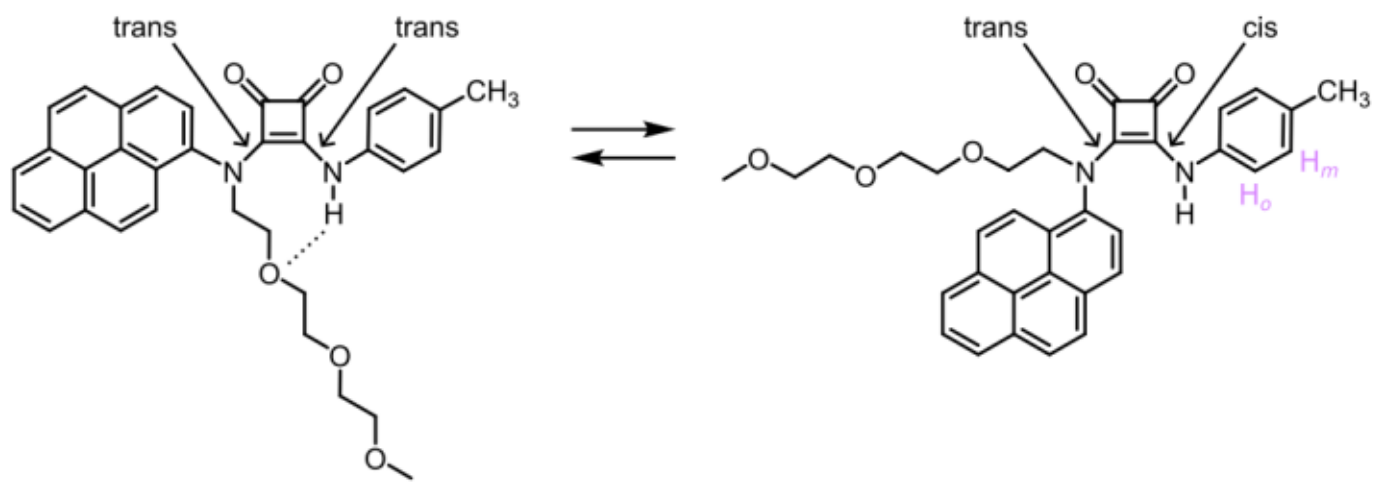

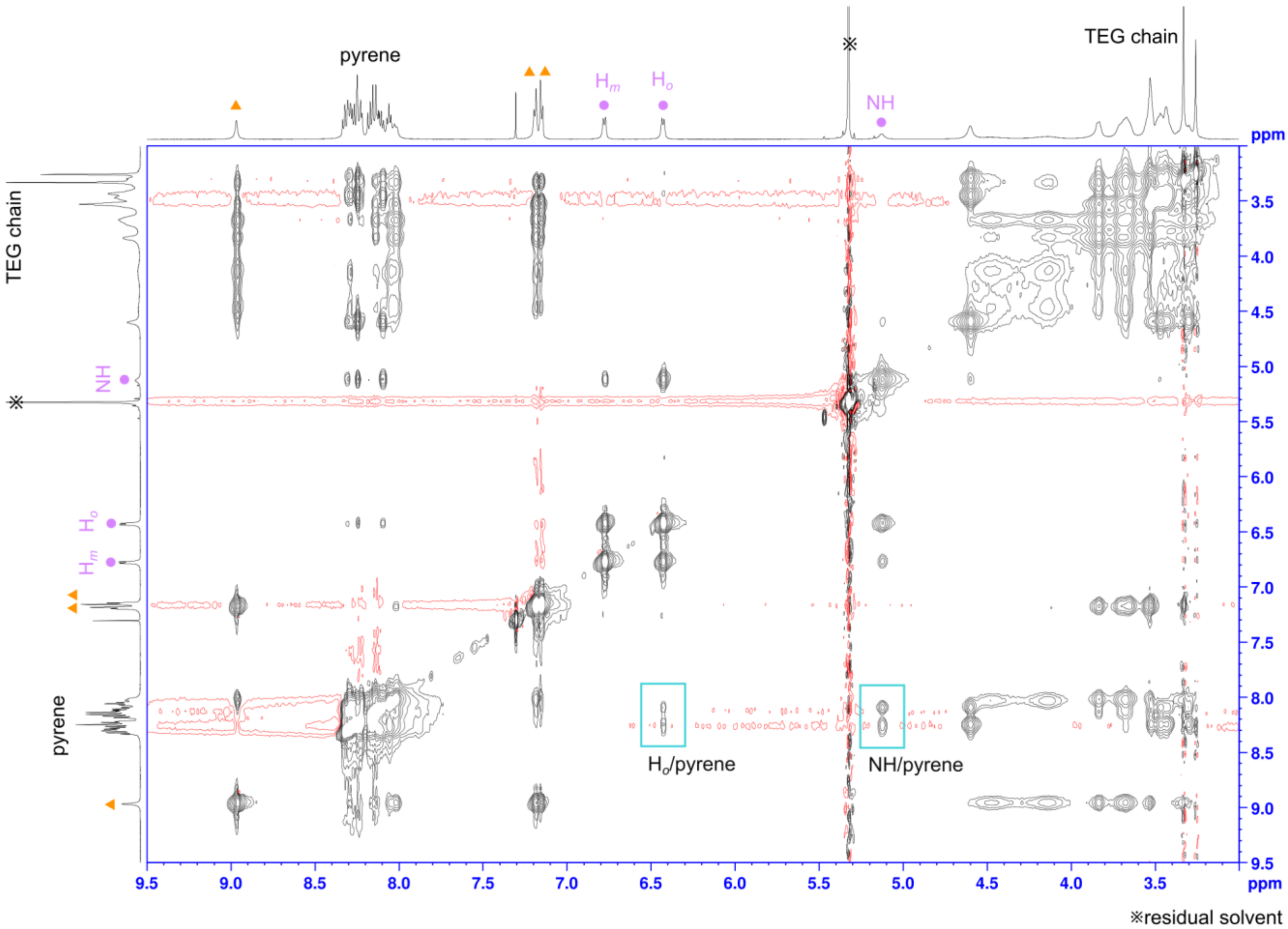

Figure S3. NOESY spectra of **1c** in CD_2Cl_2 at 183 K (600 MHz, 10 mM)

4.2. Computational methods and cartesian coordinates

Prediction of ^{1}H chemical shifts of **1c**

The initial molecular structures used for the calculations were constructed using Spartan'24 (Wavefunction, Inc.). Calculations for each conformer (cis-cis, cis-trans, trans-cis, trans-trans) were performed with Spartan'16 employing density functional theory (DFT) at the B3LYP l level of theory. Geometry optimizations were carried out in the gas phase using the 6-31G(d,p) basis set.

Subsequently, NMR shielding constants were calculated at the same level of theory (DFT/B3LYP with the 6-31G(d,p) basis set). The ^{1}H-NMR spectra were predicted from the calculated shielding tensors, and the corresponding chemical shifts and coupling constants were derived.

The relative energies of the conformers were further evaluated using the Generate Conformations tool implemented in Discovery Studio 2021 (BIOVIA, Dassault Systèms). Conformational searches were conducted using the FAST algorism with an energy threshold set to 100 kcal/mol. Among the generated conformations, the lowest-energy structure was defined as the reference (ΔE = 0 kcal/mol). The relative energy (ΔE) of each conformer was calculated with respect to this reference structure. Conformations with relative energies exceeding 100 kcal/mol were excluded from further analysis.

(*cis*, *cis*)-**1c** (0.0 kcal/mol)

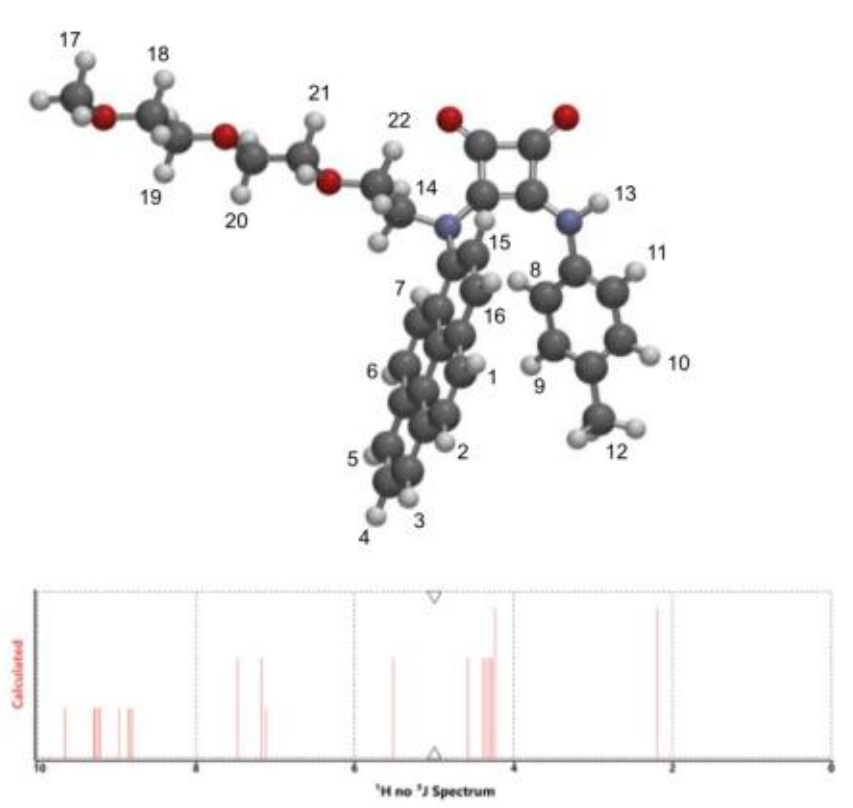

No.	Chemical Shift (ppm)	^3J (Hz)
1	8.83	^3J = 8.8
2	8.95	^3J = 9.0
3	9.20	^3J = 9.2
4	9.24	^3J = 9.2
5	9.27	^3J = 9.3
6	9.27	^3J = 9.3
7	9.64	^3J = 9.7
8	7.47	^3J = 7.5
9	7.17	^3J = 7.2
10	7.17	^3J = 7.2
11	7.47	^3J = 7.5
12	2.19	
12	2.19	
12	2.19	
13	7.11	
14	5.51	^3J = 5.5
14	5.51	^3J = 5.5
15	8.79	^3J = 8.8
16	8.84	^3J = 8.9
17	4.24	
17	4.24	
17	4.24	
18	4.28	^3J = 4.3
18	4.28	^3J = 4.3
19	4.39	^3J = 4.4
19	4.39	^3J = 4.4
20	4.35	^3J = 4.4
20	4.35	^3J = 4.4
21	4.31	^3J = 4.3
21	4.31	^3J = 4.3
22	4.58	^3J = 4.6
22	4.58	^3J = 4.6

(*trans*, *cis*)-**1c** (+5.7 kcal/mol)

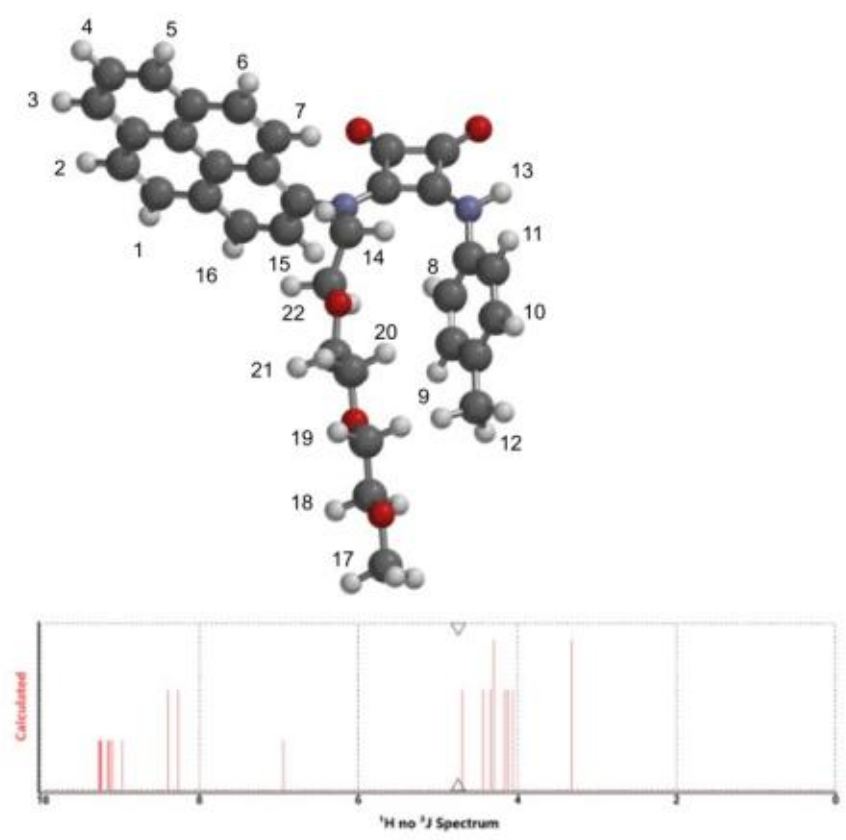

No.	Chemical Shift (ppm)	^3J (Hz)
1	9.08	^3J = 9.1
2	9.11	^3J = 9.1
3	9.26	^3J = 9.3
4	9.23	^3J = 9.2
5	9.24	^3J = 9.3
6	9.14	^3J = 9.2
7	9.23	^3J = 9.2
8	8.26	^3J = 8.3
9	8.39	^3J = 8.4
10	8.39	^3J = 8.4
11	8.26	^3J = 8.3
12	3.31	
12	3.31	
12	3.31	
13	6.94	
14	4.70	^3J = 4.7
14	4.70	^3J = 4.7
15	8.97	^3J = 9.0
16	9.13	^3J = 9.1
17	4.30	
17	4.30	
17	4.30	
18	4.34	^3J = 4.3
18	4.34	^3J = 4.3
19	4.43	^3J = 4.4
19	4.43	^3J = 4.4
20	4.17	^3J = 4.2
20	4.17	^3J = 4.2
21	4.12	^3J = 4.1
21	4.12	^3J = 4.1
22	4.06	^3J = 4.1
22	4.06	^3J = 4.1

(*cis*, *trans*)-**1c** (−4.7 kcal/mol)

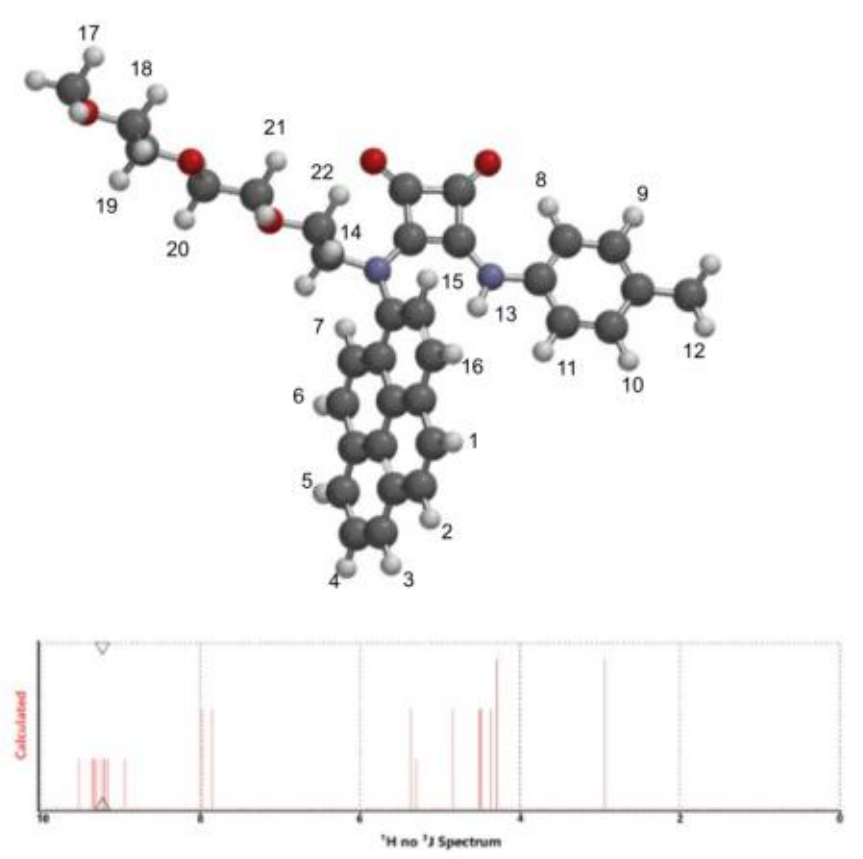

No.	Chemical Shift (ppm)	^3J (Hz)
1	9.14	^3J = 9.2
2	9.22	^3J = 9.2
3	9.35	^3J = 9.4
4	9.33	^3J = 9.3
5	9.35	^3J = 9.4
6	9.29	^3J = 9.3
7	9.51	^3J = 9.5
8	7.97	^3J = 8.0
9	7.85	^3J = 7.9
10	7.85	^3J = 7.9
11	7.97	^3J = 8.0
12	2.95	
12	2.95	
12	2.95	
13	5.30	
14	5.37	^3J = 5.4
14	5.37	^3J = 5.4
15	8.95	^3J = 9.0
16	9.19	^3J = 9.2
17	4.29	
17	4.29	
17	4.29	
18	4.36	^3J = 4.3
18	4.36	^3J = 4.3
19	4.49	^3J = 4.5
19	4.49	^3J = 4.5
20	4.50	^3J = 4.6
20	4.50	^3J = 4.6
21	4.51	^3J = 4.6
21	4.51	^3J = 4.6
22	4.85	^3J = 4.8
22	4.85	^3J = 4.8

(*trans*, *trans*)-**1c** (−4.0 kcal/mol)

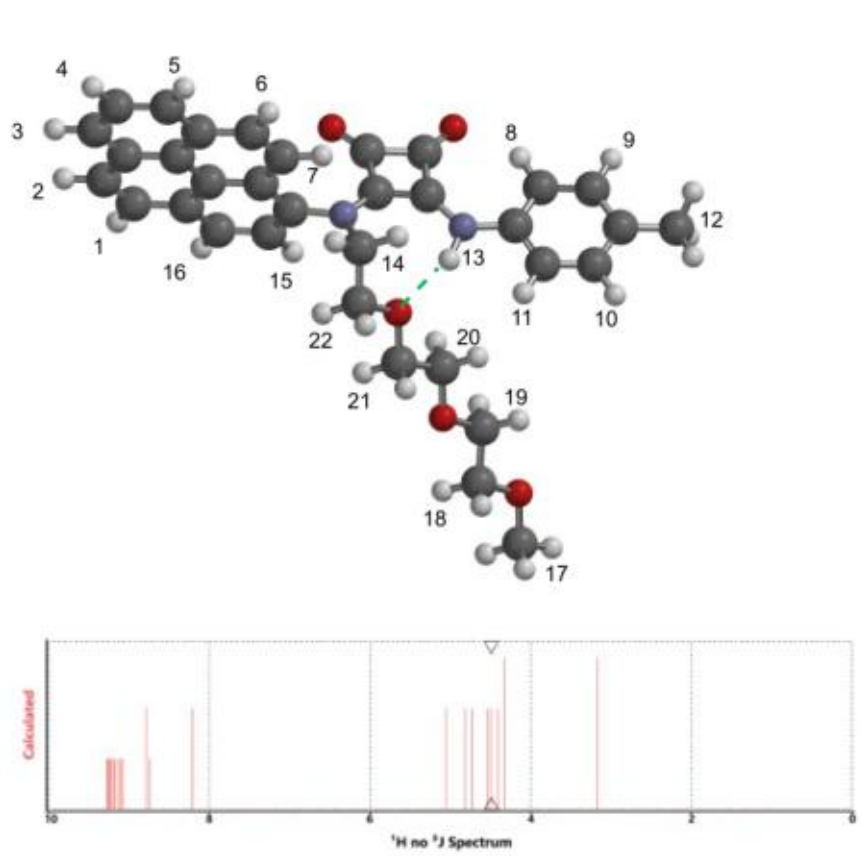

No.	Chemical Shift (ppm)	^3J (Hz)
1	9.10	^3J = 9.1
2	9.11	^3J = 9.1
3	9.27	^3J = 9.3
4	9.24	^3J = 9.2
5	9.24	^3J = 9.3
6	9.16	^3J = 9.2
7	9.22	^3J = 9.2
8	8.77	^3J = 8.8
9	8.20	^3J = 8.2
10	8.20	^3J = 8.2
11	8.77	^3J = 8.8
12	3.17	
12	3.17	
12	3.17	
13	8.74	
14	5.06	^3J = 5.1
14	5.06	^3J = 5.1
15	9.06	^3J = 9.1
16	9.18	^3J = 9.2
17	4.33	
17	4.33	
17	4.33	
18	4.41	^3J = 4.4
18	4.41	^3J = 4.4
19	4.55	^3J = 4.6
19	4.55	^3J =4.6
20	4.82	^3J = 4.8
20	4.82	^3J = 4.8
21	4.73	^3J = 4.7
21	4.73	^3J = 4.7
22	4.50	^3J = 4.5
22	4.50	^3J = 4.5

Figure S4. DFT calculation of chemical shifts of the four conformers of **1c** and the relative energies

(*cis, cis*)-**1c**

E_{DFT} = -1800.524996 a.u.

No.	Atom Name	Elemen-t	X (Å)	Y (Å)	Z (Å)
1	C1	C	-0.711	-4.755	-2.303
2	O2	O	-0.849	-5.673	-3.085
3	O1	O	-2.617	-4.049	-0.730
4	C10	C	3.085	2.428	-2.012
5	H10	H	3.035	2.758	-3.045
6	C2	C	-1.525	-3.982	-1.253
7	C11	C	3.873	3.082	-1.115
8	H11	H	4.458	3.944	-1.424
9	C12	C	3.957	2.653	0.253
10	C13	C	4.770	3.307	1.193
11	H13	H	5.349	4.172	0.879
12	C14	C	4.840	2.861	2.511
13	H14	H	5.474	3.380	3.223
14	C15	C	4.105	1.750	2.921
15	H15	H	4.168	1.403	3.949
16	C16	C	3.276	1.066	2.018
17	C17	C	2.521	-0.094	2.397
18	H17	H	2.616	-0.463	3.414
19	C18	C	1.708	-0.734	1.511
20	H18	H	1.171	-1.625	1.818
21	C19	C	1.564	-0.274	0.157
22	C20	C	2.345	0.846	-0.266
23	C21	C	3.190	1.521	0.667
24	C22	C	2.878	-3.441	-2.182
25	C23	C	3.127	-3.191	-0.829
26	H23	H	2.338	-3.325	-0.098
27	C24	C	4.397	-2.790	-0.421
28	H24	H	4.571	-2.594	0.633
29	C25	C	5.452	-2.650	-1.332
30	C26	C	5.189	-2.928	-2.679
31	H26	H	5.988	-2.838	-3.411
32	C27	C	3.921	-3.310	-3.107
33	H27	H	3.734	-3.507	-4.159
34	C28	C	6.819	-2.198	-0.876
35	H28	H	7.060	-2.594	0.115
36	H28	H	6.875	-1.104	-0.809
37	H28	H	7.600	-2.523	-1.569
38	N1	N	-0.179	-1.943	-0.368
39	N2	N	1.614	-3.877	-2.641
40	H1A	H	1.631	-4.553	-3.399
41	C3	C	-0.343	-3.065	-1.121
42	C4	C	0.412	-3.831	-2.026
43	C5	C	-1.288	-1.564	0.541
44	H5A	H	-1.722	-2.474	0.955
45	H5C	H	-0.877	-0.970	1.358
46	C6	C	0.710	-0.895	-0.783
47	C7	C	0.686	-0.457	-2.112
48	H7	H	0.022	-0.949	-2.815
49	C8	C	1.474	0.608	-2.529
50	H8	H	1.434	0.936	-3.564
51	C9	C	2.296	1.292	-1.622
52	C29	C	-2.380	-0.740	-0.143
53	O3	O	-3.200	-0.232	0.892
54	C30	C	-4.279	0.555	0.421
55	C31	C	-5.028	1.070	1.641
56	O4	O	-6.062	1.918	1.181
57	C32	C	-6.793	2.523	2.231
58	C33	C	-7.806	3.467	1.603
59	O5	O	-8.515	4.090	2.656
60	C34	C	-9.504	4.985	2.197
61	H22	H	-10.261	4.477	1.578
62	H21	H	-9.994	5.407	3.077
63	H19	H	-9.071	5.806	1.603
64	H12	H	-7.281	4.208	0.980
65	H9	H	-7.314	1.769	2.842
66	H16	H	-6.127	3.089	2.901
67	H3	H	-4.329	1.616	2.294
68	H6	H	-5.430	0.224	2.219
69	H5	H	-4.953	-0.035	-0.218
70	H20	H	-8.485	2.902	0.944
71	H2	H	-1.931	0.083	-0.722
72	H4	H	-2.959	-1.374	-0.831
73	H1	H	-3.917	1.409	-0.173

(*cis, trans*)-**1c**

E_{DFT} = -1800.532449 a.u.

No.	Atom Name	Element	X (Å)	Y (Å)	Z (Å)
1	C1	C	-0.728	-4.087	-0.998
2	O2	O	-0.941	-5.173	-1.496
3	O1	O	-2.476	-3.206	0.654
4	C10	C	3.962	2.480	-1.549
5	H10	H	3.866	2.885	-2.552
6	C2	C	-1.449	-3.177	0.003
7	C11	C	5.024	2.816	-0.766
8	H11	H	5.787	3.495	-1.138
9	C12	C	5.170	2.286	0.561
10	C13	C	6.262	2.613	1.381

11	H13	H	7.021	3.292	1.005
12	C14	C	6.378	2.075	2.662
13	H14	H	7.231	2.339	3.281
14	C15	C	5.413	1.199	3.153
15	H15	H	5.513	0.780	4.150
16	C16	C	4.302	0.845	2.370
17	C17	C	3.295	-0.066	2.834
18	H17	H	3.410	-0.503	3.823
19	C18	C	2.222	-0.397	2.064
20	H18	H	1.486	-1.105	2.427
21	C19	C	2.051	0.161	0.750
22	C20	C	3.050	1.050	0.246
23	C21	C	4.172	1.394	1.059
24	C22	C	2.208	-3.676	-2.636
25	C23	C	1.740	-4.928	-3.058
26	H23	H	0.793	-5.313	-2.694
27	C24	C	2.503	-5.673	-3.953
28	H24	H	2.124	-6.641	-4.271
29	C25	C	3.731	-5.217	-4.452
30	C26	C	4.176	-3.963	-4.021
31	H26	H	5.122	-3.572	-4.387
32	C27	C	3.431	-3.200	-3.126
33	H27	H	3.800	-2.227	-2.808
34	C28	C	4.547	-6.058	-5.405
35	H28	H	3.925	-6.475	-6.205
36	H28	H	5.018	-6.904	-4.890
37	H28	H	5.345	-5.472	-5.871
38	N1	N	-0.097	-1.008	0.415
39	N2	N	1.511	-2.847	-1.730
40	H1A	H	1.970	-1.967	-1.536
41	C3	C	-0.340	-2.209	-0.166
42	C4	C	0.338	-3.035	-1.081
43	C5	C	-1.087	-0.453	1.359
44	H5A	H	-1.517	-1.272	1.939
45	H5C	H	-0.568	0.229	2.037
46	C6	C	0.946	-0.152	-0.075
47	C7	C	0.858	0.373	-1.370
48	H7	H	0.000	0.114	-1.983
49	C8	C	1.842	1.222	-1.867
50	H8	H	1.753	1.622	-2.873
51	C9	C	2.941	1.587	-1.075
52	C29	C	-2.212	0.301	0.649
53	O3	O	-3.000	0.905	1.654
54	C30	C	-4.124	1.598	1.140
55	C31	C	-4.929	2.113	2.324
56	O4	O	-6.018	2.846	1.799
57	C32	C	-6.891	3.351	2.791
58	C33	C	-7.976	4.144	2.079
59	O5	O	-8.860	4.639	3.064
60	C34	C	-9.923	5.391	2.520
61	H22	H	-10.536	4.793	1.828
62	H21	H	-10.549	5.719	3.353
63	H19	H	-9.561	6.279	1.977
64	H12	H	-7.516	4.965	1.504
65	H9	H	-7.350	2.535	3.372
66	H16	H	-6.357	4.004	3.499
67	H3	H	-4.291	2.745	2.962
68	H6	H	-5.274	1.264	2.934
69	H5	H	-4.751	0.935	0.526
70	H20	H	-8.501	3.492	1.362
71	H2	H	-1.793	1.061	-0.032
72	H4	H	-2.808	-0.405	0.052
73	H1	H	-3.813	2.445	0.508

(*trans, cis*)-**1c**

E_{DFT} = -1800.51596 a.u.

No.	Atom Name	Element	X (Å)	Y (Å)	Z (Å)
1	C1	C	-2.464	-4.722	0.948
2	O2	O	-2.598	-5.896	1.228
3	O1	O	-4.491	-3.276	0.298
4	C10	C	-6.014	2.215	-1.014
5	H10	H	-6.462	2.706	-0.155
6	C2	C	-3.324	-3.531	0.480
7	C11	C	-6.425	2.518	-2.276
8	H11	H	-7.206	3.257	-2.437
9	C12	C	-5.850	1.871	-3.423
10	C13	C	-6.261	2.166	-4.732
11	H13	H	-7.037	2.911	-4.887
12	C14	C	-5.689	1.515	-5.824
13	H14	H	-6.021	1.756	-6.829
14	C15	C	-4.699	0.554	-5.634
15	H15	H	-4.262	0.044	-6.489
16	C16	C	-4.254	0.227	-4.343
17	C17	C	-3.251	-0.771	-4.107
18	H17	H	-2.842	-1.305	-4.960
19	C18	C	-2.822	-1.065	-2.849
20	H18	H	-2.086	-1.847	-2.697
21	C19	C	-3.351	-0.381	-1.700
22	C20	C	-4.384	0.587	-1.898
23	C21	C	-4.828	0.895	-3.219
24	C22	C	1.013	-3.374	1.693

25	C23	C	0.699	-2.370	2.618
26	H23	H	-0.336	-2.210	2.905
27	C24	C	1.714	-1.603	3.185
28	H24	H	1.454	-0.833	3.908
29	C25	C	3.063	-1.820	2.868
30	C26	C	3.360	-2.847	1.963
31	H26	H	4.396	-3.046	1.703
32	C27	C	2.354	-3.613	1.375
33	H27	H	2.603	-4.393	0.662
34	C28	C	4.149	-0.957	3.465
35	H28	H	3.977	-0.782	4.533
36	H28	H	4.189	0.026	2.979
37	H28	H	5.133	-1.422	3.356
38	N1	N	-1.800	-1.521	-0.138
39	N2	N	0.005	-4.187	1.107
40	H1A	H	0.107	-5.186	1.254
41	C3	C	-2.034	-2.756	0.360
42	C4	C	-1.288	-3.833	0.862
43	C5	C	-0.455	-1.070	-0.524
44	H5A	H	0.251	-1.877	-0.327
45	H5C	H	-0.425	-0.862	-1.598
46	C6	C	-2.911	-0.633	-0.383
47	C7	C	-3.515	0.003	0.703
48	H7	H	-3.165	-0.228	1.704
49	C8	C	-4.541	0.920	0.513
50	H8	H	-5.001	1.404	1.370
51	C9	C	-4.982	1.244	-0.779
52	C29	C	0.006	0.173	0.235
53	O3	O	1.346	0.398	-0.160
54	C30	C	2.032	1.330	0.655
55	C31	C	3.485	1.334	0.206
56	O4	O	4.225	2.086	1.153
57	C32	C	5.586	2.247	0.793
58	C33	C	6.261	3.058	1.888
59	O5	O	7.618	3.208	1.525
60	C34	C	8.355	3.967	2.461
61	H22	H	8.346	3.504	3.460
62	H21	H	9.385	4.014	2.102
63	H19	H	7.963	4.992	2.554
64	H12	H	5.761	4.036	1.987
65	H9	H	6.087	1.272	0.690
66	H16	H	5.679	2.773	-0.170
67	H3	H	3.574	1.771	-0.800
68	H6	H	3.848	0.296	0.154
69	H5	H	1.982	1.032	1.713
70	H20	H	6.160	2.537	2.854
71	H2	H	-0.629	1.041	-0.001
72	H4	H	-0.059	-0.009	1.317
73	H1	H	1.597	2.338	0.569

(*trans*, *trans*)-**1c**

E_{DFT} = -1800.531432 a.u.

No.	Atom Name	Element	X (Å)	Y (Å)	Z (Å)
1	C1	C	-1.875	-1.930	-2.197
2	O2	O	-2.079	-2.884	-2.920
3	O1	O	-3.884	-1.027	-0.899
4	C10	C	-5.159	2.594	3.557
5	H10	H	-5.271	2.006	4.464
6	C2	C	-2.735	-1.047	-1.282
7	C11	C	-5.892	3.726	3.374
8	H11	H	-6.596	4.054	4.135
9	C12	C	-5.765	4.512	2.178
10	C13	C	-6.514	5.680	1.966
11	H13	H	-7.213	6.006	2.732
12	C14	C	-6.374	6.415	0.790
13	H14	H	-6.964	7.315	0.644
14	C15	C	-5.486	6.001	-0.200
15	H15	H	-5.388	6.574	-1.118
16	C16	C	-4.714	4.841	-0.032
17	C17	C	-3.805	4.371	-1.038
18	H17	H	-3.735	4.926	-1.970
19	C18	C	-3.058	3.247	-0.855
20	H18	H	-2.408	2.894	-1.647
21	C19	C	-3.141	2.486	0.362
22	C20	C	-4.065	2.906	1.368
23	C21	C	-4.845	4.085	1.172
24	C22	C	1.397	-1.760	-3.004
25	C23	C	0.856	-2.694	-3.900
26	H23	H	-0.205	-2.918	-3.883
27	C24	C	1.697	-3.350	-4.794
28	H24	H	1.259	-4.072	-5.479
29	C25	C	3.079	-3.115	-4.836
30	C26	C	3.598	-2.178	-3.937
31	H26	H	4.663	-1.962	-3.942
32	C27	C	2.776	-1.505	-3.036
33	H27	H	3.205	-0.766	-2.364
34	C28	C	3.967	-3.855	-5.807
35	H28	H	3.565	-3.823	-6.825
36	H28	H	4.066	-4.913	-5.534
37	H28	H	4.974	-3.427	-5.832
38	N1	N	-1.360	0.932	-0.349

39	N2	N	0.627	-1.034	-2.072	57	C32	C	4.436	-3.191	1.867
40	H1A	H	1.166	-0.510	-1.388	58	C33	C	5.410	-3.445	3.008
41	C3	C	-1.516	-0.229	-1.035	59	O5	O	6.034	-4.687	2.760
42	C4	C	-0.709	-1.060	-1.834	60	C34	C	6.973	-5.037	3.756
43	C5	C	-0.147	1.756	-0.438	61	H22	H	6.502	-5.130	4.747
44	H5A	H	0.308	1.611	-1.422	62	H21	H	7.399	-6.002	3.475
45	H5C	H	-0.447	2.806	-0.374	63	H19	H	7.785	-4.296	3.829
46	C6	C	-2.364	1.332	0.601	64	H12	H	6.148	-2.627	3.052
47	C7	C	-2.538	0.587	1.769	65	H9	H	3.683	-3.993	1.832
48	H7	H	-1.940	-0.308	1.913	66	H16	H	4.977	-3.200	0.909
49	C8	C	-3.458	0.979	2.734	67	H3	H	3.379	-1.551	0.100
50	H8	H	-3.580	0.387	3.636	68	H6	H	2.067	-2.290	1.041
51	C9	C	-4.223	2.143	2.566	69	H5	H	1.888	-0.217	2.433
52	C29	C	0.880	1.486	0.656	70	H20	H	4.867	-3.452	3.967
53	O3	O	1.447	0.202	0.444	71	H2	H	1.661	2.261	0.620
54	C30	C	2.376	-0.190	1.448	72	H4	H	0.401	1.534	1.645
55	C31	C	2.901	-1.573	1.093	73	H1	H	3.211	0.524	1.504
56	O4	O	3.828	-1.931	2.096						

4.3. Temperature-dependent ^{1}H NMR spectra

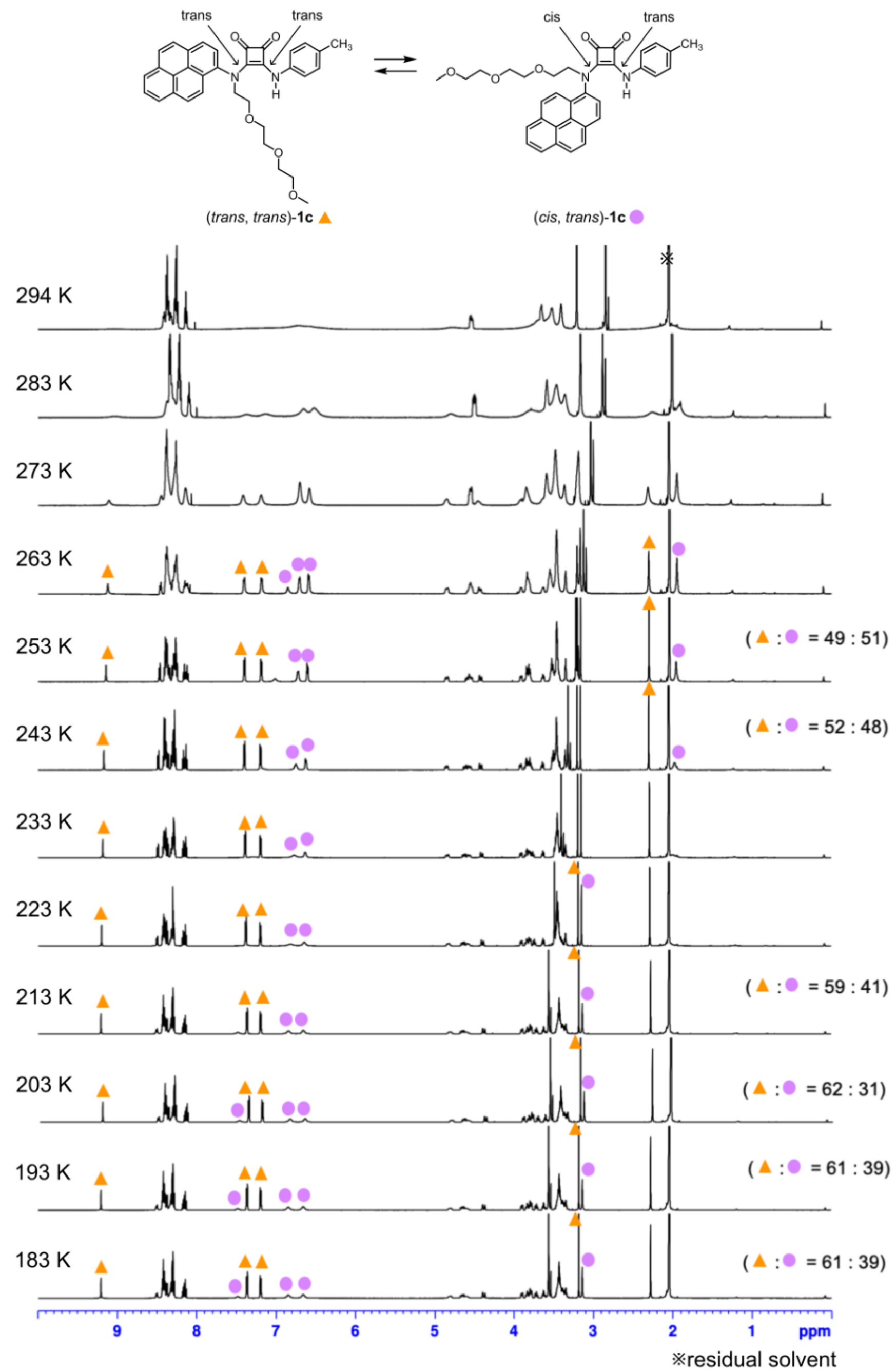

Figure S5. Temperature-dependent ^{1}H NMR spectra of **1c** in acetone-d_6 (600 MHz, 10 mM)

5. Temperature-dependent ^{1}H NMR spectra of compound 1d

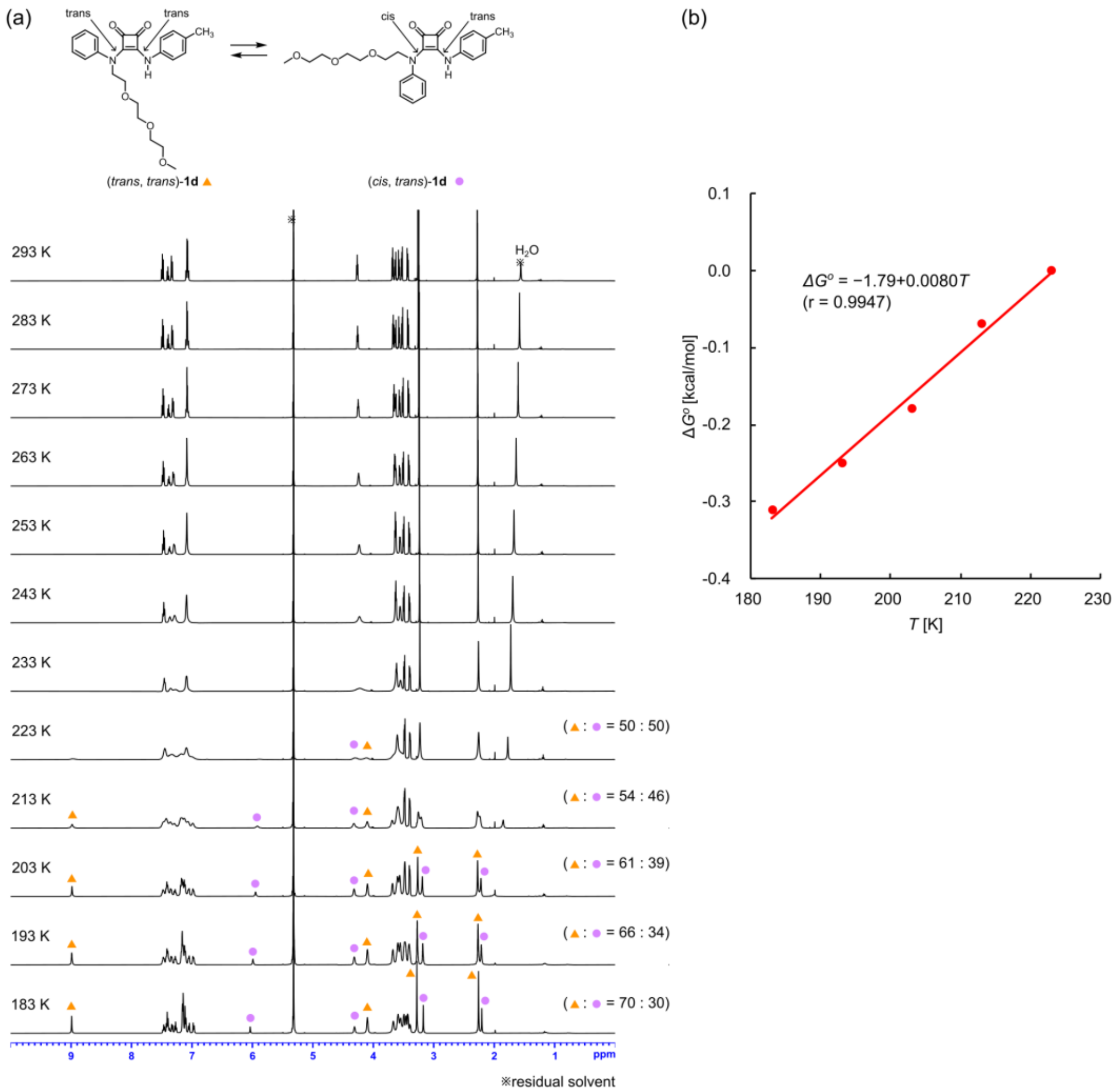

Figure S6. (a) Temperature-dependent ^{1}H NMR spectra of **1d** in CD_2Cl_2 and (b) van't Hoff plot (500 MHz, 10 mM)
